# Generalizing soil properties in geographic space: Approaches used and ways forward

**DOI:** 10.1371/journal.pone.0208823

**Published:** 2018-12-21

**Authors:** Carmen Cianfrani, Aline Buri, Eric Verrecchia, Antoine Guisan

**Affiliations:** 1 Institute of Earth Surface Dynamics (IDYST), University of Lausanne (UNIL), Lausanne, Switzerland; 2 Department of Ecology and Evolution (DEE), University of Lausanne (UNIL), Lausanne, Switzerland; The University of Sydney, AUSTRALIA

## Abstract

Soil is one of the most complex systems on Earth, functioning at the interface between the lithosphere, biosphere, hydrosphere, and atmosphere and generating a multitude of functions. Moreover, soil constitutes the belowground environment from which plants capture water and nutrients. Despite their great importance, soil properties are often not sufficiently considered in other disciplines, especially in spatial studies of plant distributions. Most soil properties are available as point data and, to be used in spatial analyses, need to be generalised over entire regions (i.e. digital soil mapping). Three categories of statistical approaches can be used for such purpose: geostatistical approaches (GSA), predictive-statistical approaches (PSA), and hybrid approaches (HA) that combine the two previous ones. How then to choose the best approach in a given soil study context? Does it depend on the soil properties to be spatialized, the study area’s characteristics, and/or the availability of soil data? The main aims of this study was to review the use of these three approaches to derive maps of soil properties in relation to the soil parameters, the study area characteristics, and the number of soil samples. We evidenced that the approaches that tend to show the best performance for spatializing soil properties were not necessarily the ones most used in practice. Although PSA was the most widely used, it tended to be outperformed by HA in many cases, but the latter was far less used. However, as the study settings were not always properly described and not all situations were represented in the set of papers analysed, more comparative studies would be needed across a wider range of regions, soil properties, and spatial scales to provide robust conclusions on the best spatialization methods in a specific context.

## Introduction

Soil, an emergent property resulting from the interactions between the lithosphere, biosphere, hydrosphere, and atmosphere, is among the most complex components on Earth and is a crucial component of all terrestrial ecosystems [1, 2]. Soil generates a multitude of functions, as it forms a carbon pool, sustains biomass production, and stores, filters, and transforms nutrients [3, 4]. It also constitutes the belowground environment from which plants capture water and nutrients [5] and supports an amazingly rich belowground biodiversity [6]. All soils contain rocks, mineral particles, organic matter, water, and air, which combinations determines the soil properties, i.e., its texture, structure, porosity, chemistry, and colour [1]. Despite their importance, soil properties are rarely considered in spatial studies of biodiversity, ecosystems and their services [3]. For instance, soil properties are only occasionally included in studies of plant distributions, even considering its importance for defining the environmental niche of plants [7]. There are various potential reasons for this. Field measurements of soil related factors are time consuming and costly to perform. Furthermore, most soil properties are sampled at specific locations, while to be used in other disciplines of natural sciences, they often need to be generalised over a whole geographical extent, which is challenging because of the complexity of soil systems. Meeting this challenge requires combining field surveys, the most recent numeric data, remote sensing and geographic information technology, and advanced spatial methods in the same area [7]. There are many techniques available to generalize soil properties from point data to continuous maps (i.e. digital soil mapping, DSM; [8, 9]. These spatialization techniques (in general, not only for soils) can be classified into three different types of approaches: geostatistical approaches (GSA), predictive-statistical approaches (PSA), and hybrid approaches (HA) [10].

Geostatistical approaches (GSA) deal primarily with the spatial variation of observation points compared to the neighbourhood of each point. Ordinary kriging, for instance, uses variograms (a special type of autocorrelograms) to compute functions of distance and variation, assuming some spatial autocorrelation in the data. However, the latter assumption can sometimes be poor in complex terrain characterized by abrupt changes in soil-forming factors [8, 9]. In such a landscape, the amount of data required to use geostatistical approaches to derive maps would prove too difficult and costly to collect, given the strict sampling protocol required to characterize spatial dependence [9].

Predictive statistical approaches (PSA) exploit the statistical relationship between soil properties and a suite of quantifiable environmental variables across a set of observation points. Because it does not explicitly consider the spatial arrangement of points, it is sometimes considered to be ‘aspatial’. Multiple linear regression and related techniques (e.g., generalised linear or additive models such as GLM or GAM) have largely been used to derive these models because of their simplicity, computational efficiency, and straightforward interpretation [8]. However, many other predictive techniques exist [8, 10], e.g., based on recursive partitioning (i.e. decision tree approaches; e.g. [9, 11] or machine learning algorithms [12], which have better potential for accounting for the complex relationships between soil-forming factors and soil properties. For instance, the random forest approach [13, 14] has several advantages that made it promoted as a favourable method for soil predictions. It was reported as being suitable for datasets with many predictor features but with only a few samples, to be robust to noise, and to require little fine tuning of the parameters to produce good predictions [15]. Random forest aggregates multiple predictions based on changes in the training dataset through resampling iterations [16].

In recent years, there has been increasing interest in hybrid approaches (HA) that combine predictive and geostatistical methods [17–19]. For example, regression-kriging combines a regression of the response variable (here, soil) on explanatory predictor variables (here, environmental descriptors, such as those derived from a digital elevation model, from remote sensing imagery, or from thematic maps) with kriging of the regression residuals. It is mathematically equivalent to the interpolation method variously called universal kriging and kriging with external drift, where environmental predictors are used directly to determine the kriging weights.

During the last few years, a large amount of studies has been carried out to map soil properties for many landscapes and as a function of soil data availability (see the review by Grunwald [10]). However, the ways in which authors choose their approach often remained unclear. Considering the numerous spatialization approaches and techniques, identifying the one(s) that is(are) best adapted to spatializing a particular soil property in a given study area is not an easy task.

A substantial amount of reviews with different objectives have been published on digital soil mapping. Among the oldest ones, McBratney [20] proposed an overview of the research in digital soil mapping (DSM). McBratney et al. [8] as well as Grunwald [9] then reviewed approaches used to incorporate environmental GIS data in soil mapping. More recently, Grunwald [10] assessed the usefulness of recent digital soil mapping and modelling (DSMM; hereafter simply DSM) approaches to meet specific needs at local, national, and global scales, and Grunwald et al. [21] reviewed studies on digital soil mapping at the continental scale. Minasny et al. [22] defined what constitutes digital soil mapping and reviewed several key concepts of the history of digital soil mapping. Brevik et al. [23] reviewed the accomplishments to date and discussed some ways forward in soil survey, classification, and pedological modelling. Finally, Keskin and Grunwald [24] reviewed studies using regression kriging, a special type of HA, in order to quantify the factors affecting the performance of this technique. Several of these reviews provided some history of soil mapping at their time of publication [25–27]. All of these reviews confirmed the large number of different techniques and approaches used in soil mapping and modelling, but none of them reviewed which approach was used in which context. As a matter of fact, it seems that the multitude of techniques made available in recent years has hampered the emergence of such general guidelines for digital mapping of soils and their properties.

Focusing on three specific types of approaches—geostatistical approaches (GSA), predictive-statistical approaches (PSA), and hybrid approaches (HA)—our aims were: 1) to review the methods used in digital soil mapping (and modelling) in relation to soil properties, study area characteristics, and soil sample availability; 2) to identify the best approach in each context based on the three previous factors; and finally 3) assess whether there is a concordance between the reported performance of these three approaches and the frequency at which they were used in published studies. To meet with these aims, we reviewed papers published during the period 2010–2016 in *Geoderma*, *European Journal of Soil Science*, and *Soil Tillage Research*. The selected papers were further divided into two groups: 1) papers that compared more than one different spatialization approach (i.e., GSA, PSA and HA), which we then used to compare the modelling performance of the different approaches within the same study, and 2) papers that considered only one type of DSM approach, in order to identify which of the spatialization approaches was the most used for which soil properties and in which types of study areas.

## Methods

**Fig 1:**
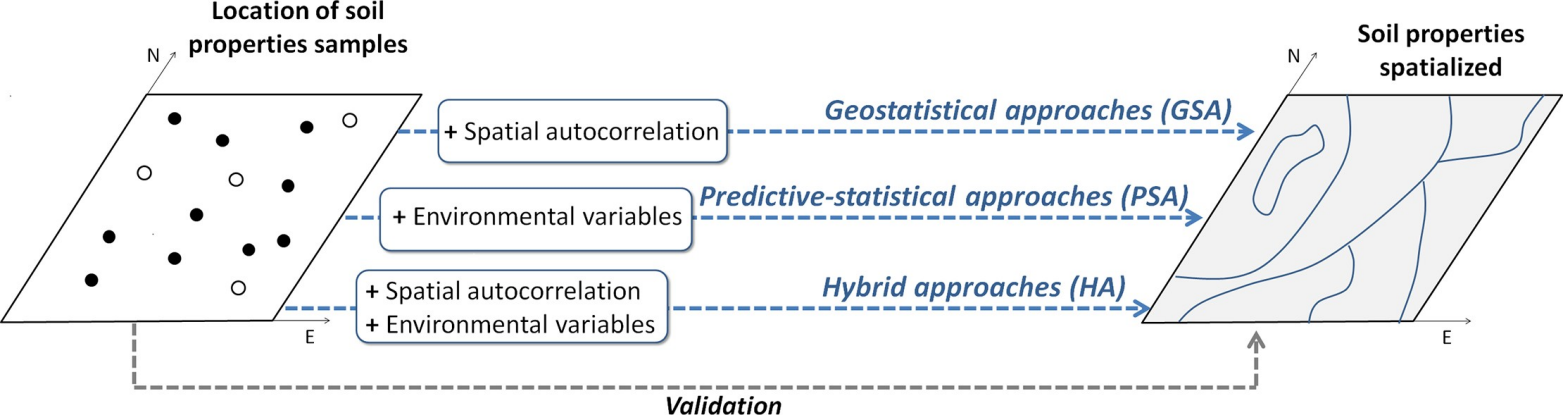
Flowchart of the used approaches, showing how maps of soil properties can be generated from point measurements.

We performed a web search to extract original articles dealing with the spatial prediction of soil properties (excluding reviews, opinions, and perspectives). The target of the search was to review articles published in high-quality soil journals over the period of 2010–2016. The following soil journals were selected according to an ISI impact factor greater than 3 during the period 2014–2015 (http://www.scimagojr.com): *Geoderma*, *European Journal of Soil Science*, and *Soil Tillage Research*. The search was performed using the following query: “(´digital mapping*´ OR ´mapping*´ OR ´prediction*´ OR ´spatial prediction*´ OR ´spatial distribution*´ OR ´interpolation*´) AND (´soil properties´ OR ´edaphic factors*´)”, in the ISI Web of Science (WoS) website. The search resulted in 373 papers (S1 Appendix), but interest was focused only on those studies that attempted to spatialize soil properties and validated their prediction using an independent dataset (Fig 1), whereas publications focused on experimental and analytical laboratory/plots or studies were not considered in the survey. This resulted in a final selection of 105 papers (S1 Table and S2 Table) out of the 373 from our initial search.

**Fig 2:**
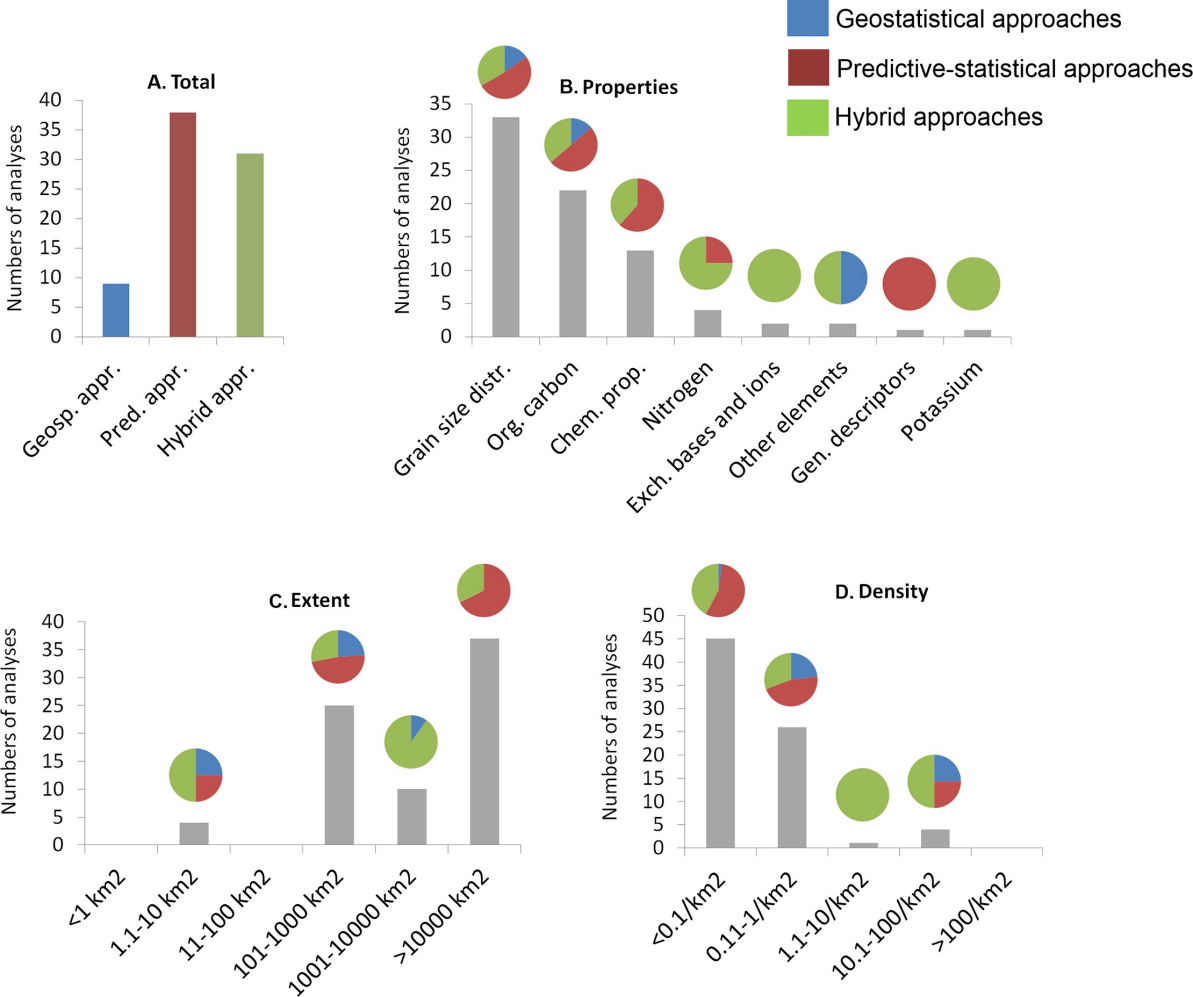
Representation of the different categories across the reviewed papers. **—A**: total number of studies for each category; **B**: soil properties; **C**: extent of study area; and **D**: density of soil samples in the study area. Pie charts represent the number of analyses used in each approach (geostatistical, predictive statistical, and hybrid) that obtained the highest spatialization performance value.

**Fig 3:**
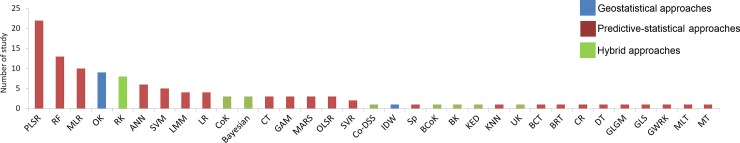
Frequency of use of the different spatialization techniques across the reviewed papers. **–**Bars represent the number of studies that used the following techniques to predict soil properties: PLSR (Partial Least Squares Regression), RF (Random Forest), MLR (Multi Linear regression), OK (Ordinary Kriging), RK (Regression Kriging), ANN (Artificial Neural Network), SVM (Support Vector Machine), LMM (Linear Mixed Model), LR (Linear Regression), CoK (Co-kriging), Bayesian (Bayesian), CT (Classification trees), GAM (Generalised Additive Model), MARS (Multivariate Adaptive Regression Splines), OLSR (Ordinary Least squares Regression), SVR (Support Vector Regression), Co-DSS (Direct Sequential Co-Simulation), IDW (Inverse Distance Weighted), SK (Simple kriging), Sp (Splines), BCok (Block-Co-Kiging), BK (Block Kriging), KED (Kriging with external drift), KNN (K-nearest neighborhood), UK (Universal kriging), BCT (Boosted classification tree), BRT (Boosted Regression Trees), CR (Cubist regression), DT (Decision tree), GLGM (Generalised linear geostatistical model), GLS (Generalised least squares), GWRK (Generalised Weighted Regression Kriging), MLT (Machine learning tree), and MT (Model Tree).

**Fig 4:**
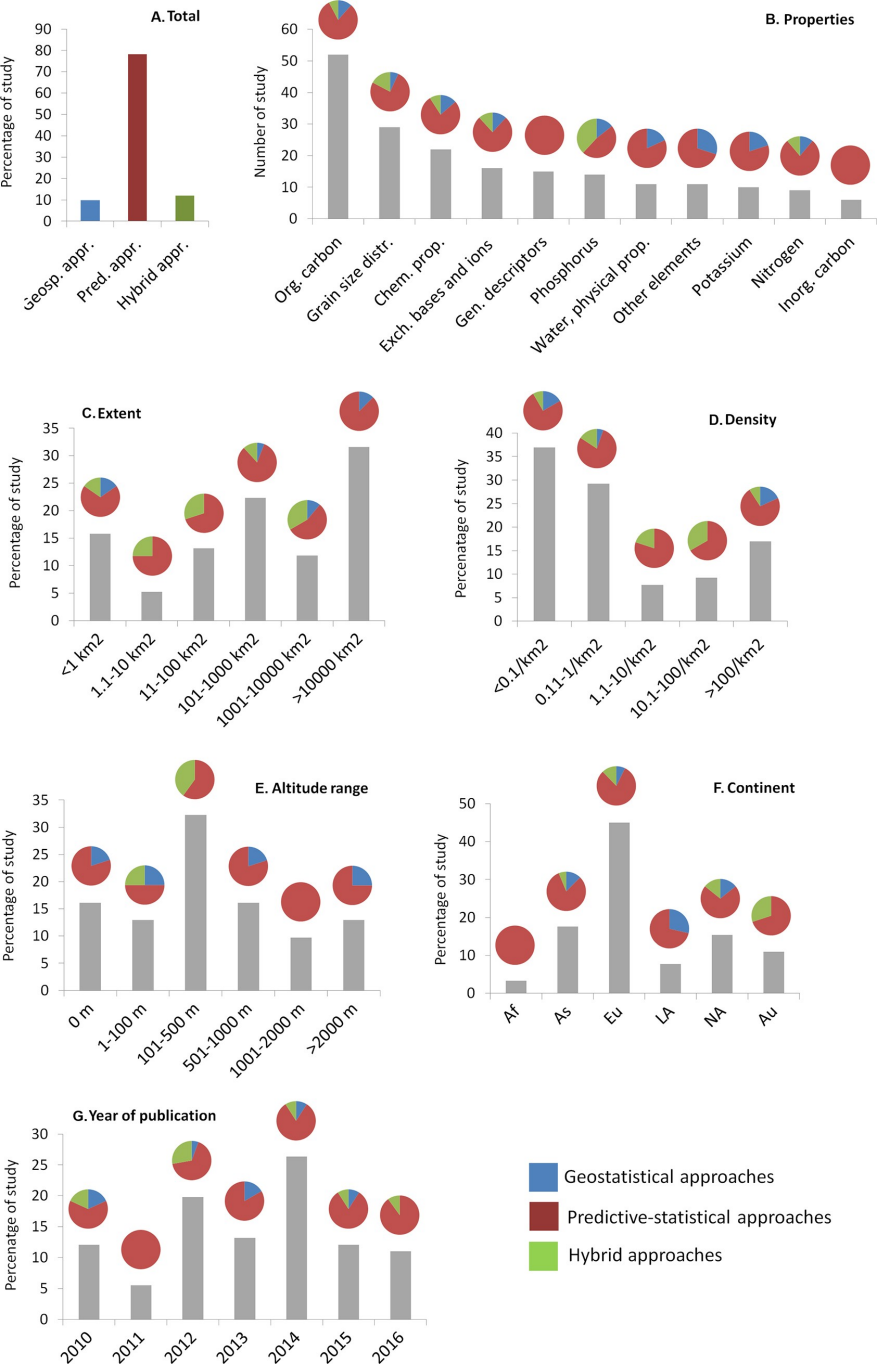
Frequency of use of the different approaches applied in the reviewed papers. Bars represent the following: **A.** The number of studies that used the geostatistical approaches, spatial-predictive approaches and hybrid approaches. **B.** Soil property classes: water and physical properties, grain size distribution, general descriptors, organic carbon, inorganic carbon, chemical properties, nitrogen, phosphorus, potassium, other elements, and exchangeable bases and associated ions (K excluded; see Table 1 for more details). **C.** Percentage of studies carried out using different densities of sample points in the study areas (n. samples/km^2^); **D.** Percentage of studies carried out in different study area extent classes (km^2^). **E.** Percentage of studies carried out in study areas with different altitudinal range (m) classes. **F.** Percentage of studies carried out on each continent: Af (*Africa*), As (*Asia*), Eu (*Europe*), LA (*Latin America*), NA (*North America*), and Au (*Australia*). **G.** Percentage of studies published in each year. The pie diagrams represent the percentage of studies that used predictive statistical, geostatistical and hybrid approaches.

For all of the 105 selected articles, the following information was recorded: techniques used for spatialization, spatialized soil properties, the number of soil samples used in the study, the altitude and extent of the study area, and the density of plots (Fig 2, Fig 3, Fig 4). The year of publication and the continent were also recorded. As introduced earlier (introduction), one of three types of soil spatialization techniques was assigned to each study: (i) *geostatistical approaches* (*GSA*), (ii) *predictive statistical approaches* (*PSA*), and (iii) *hybrid approaches* (*HA*). GSA is primarily based on the spatial configuration of samples and accounts for spatial autocorrelation; PSA is based on statistical, correlative relationships between soil properties and environmental factors; and HA combines GSA and PSA [8, 9].

Soil properties were classified into the following 11 categories (see Table 1): (i) water and physical properties, (ii) grain size distribution, general descriptors (such as the horizon depth, nature of the parental material, stoniness, or topsoil thickness), (iii) organic carbon, (iv) inorganic carbon, (v) chemical properties, (vi) nitrogen, (vii) phosphorus, (viii) potassium, (ix) exchangeable bases and associated ions (K excluded), and (x) other elements (aluminium, iron, etc.; see Table 1). Moreover, six classes of altitudinal ranges have been defined (flat areas, 1–100 m, 101–500 m, 501–1000 m, 1001–2000 m, and > 2000 m), as well as six classes of study area extent (< 1 km^2^, 1.1–10 km^2^, 11–100 km^2^, 101–100 km^2^, 1001–10000 km^2^, and > 10000 km^2^) and five classes of sample density (< 0.1/km^2^, 0.11-1/km^2^, 1.1-10/km^2^, 10.1-100/km^2^, and > 100/km^2^).

**Table 1:** Soil properties found in the reviewed papers, and their classification.

Spatialized soil property	Classes
Available water capacity, Bulk density, Moisture content (MC), Soil drainage	Water and physical properties
Total coarse fragments, Cobble, Gravel, Sand, Silt, Clay	Grain size distribution
Horizons depths, Parental material, Stoniness, Topsoil thickness, Type/class	General descriptors
Carbon stock, SOC, SOM	Organic carbon
CaCO_3_, Inorganic Carbon, MINC	Inorganic carbon
Acidity, C:N, Electrical conductivity (EC), Loss on ignition, pH, Salinity	Chemical properties
Ammonium nitrogen, Hot water extractable nitrogen, Nitrate, Nitrate nitrogen (NO_3_), Total nitrogen	Nitrogen
Available phosphorus, Phosphate (PO_4_^3−^), Phosphorus absorptive coefficient (PAC), Phosphorus pentoxide (P_2_O_5_), Total phospourus (P)	Phosphorus
K^+^, Potassium (K), Potassium oxide (K_2_O), Available potassium	Potassium
Al, Cd, Cu, Fe, Pb, Redness rating, Sulfur (S)	Other elements
Base saturation, Ca, Ca^2+^, CEC, Mg, Mg^2+^, Na^+^, Sodium (Na), Sodium absorption ratio (SAR), Sum of bases, Sum of exchangeable bases	Exchangeable bases and associated ions (K excluded)

To answer our initial question, we also split the different studies into two groups. The first group, the comparative studies, included studies that compared at least two of the different approaches (i.e., GSA, PSA, and HA). Because in some studies different soil properties and soil depths were considered, we counted the number of analyses devoted to each approach that obtained the best spatialization performance. This first group was then used to compare the modelling performance of the different approaches within the same study (Fig 2). The second group included studies that considered only one class of spatialization approach. We then used this second group to identify which of the spatialization approaches was the most used for which soil properties and in which types of study areas (Fig 4).

## Results

### The first group: Comparative studies

Twelve papers were found that used and compared different spatialization techniques belonging to more than one type of the approaches previously mentioned (i.e., GSA, PSA and HA) to spatialize soil properties across landscapes. For example, de Carvalho et al. [28] assessed the power of ordinary kriging, regression kriging, and a linear model–corresponding to GSA, HA, and PSA, respectively–to spatialize soil attributes in a tropical environment. Guo et al. [18] compared linear regression, random forest (both being PSA), and regression kriging (HA) for spatializing SOC (soil organic carbon). Hoffmann et al. [29] carried out a study in a high elevation range zone (1900 m) and a very small area (8 km^2^) using a high density of samples (47 samples/km^2^) to compare the performances of inverse distance weighted, ordinary kriging (both being GSA), block kriging, and regression kriging (both being HA; S1 Table).

Considering that some studies included in their analysis different soil properties and soil depths, we finally ended up with 78 investigations that considered analyses in the same study area but on different soil parameters. In the majority of cases, PSA showed the highest validation values (n = 38), followed by HA (n = 31). On the contrary, GSA performed less well, as it was superior to the two others approaches in only 12.5% of cases (n = 9; Fig 2B).

Specific analyses were not available or were available for only very few examples for some soil properties, characteristics of study areas, and density classes of soil samples (Fig 2). This was the case for the properties (see Table 1) ‘nitrogen’, ‘exchangeable bases and ions’, ‘other elements’, ‘general descriptors’, and ‘potassium’, as well as for study areas < 100 km^2^ and soil density classes > 1 sample per km^2^. There were also not enough studies to analyse differences in altitudinal ranges.

Generally regarding the spatialization of the categories of soil properties, the highest numbers of analyses were carried out to spatialize the properties of soil grain size distributions (n = 33), followed by organic carbon (n = 22) and chemical characteristics (n = 13; Fig 2B). Concerning the extent of the study area, the majority of analyses were performed in areas > 1000 km^2^ (n = 37), followed by areas ranging from 100 to 1000 km^2^ (n = 25) and those ranging from 1000 to 10000 km^2^ (n = 10; Fig 2C). Only two classes of sample densities were sufficiently represented, i.e., analyses using < 0.1 samples per km^2^ (n = 45) and between 0.1 and 1 sample per km^2^ (n = 26; Fig 2D).

According to the soil properties, PSA performed the best in the majority of cases, with the exceptions of nitrogen, exchange bases and ions, and potassium classes, for which the hybrid approaches proved better (however, see above; few analyses were available for these classes). GSA had the highest percentage of the best performances for the grain size distributions and organic carbon classes, respectively 15% and 14% of the analyses; however, compared to the latter, PSA remained the best in 45% of the studies for the category cited above (Fig 2A). Regarding the extent of study areas, GSA was best in 24% of analyses carried out in areas ranging from 100 to 1000 km^2^. HA performed best in 90% of the analyses carried out in areas ranging from 1000 to 10000 km^2^. Finally, PSA gave the best results in 68% of analyses carried out in large areas > 10000 km^2^ (Fig 2C). Concerning the densities of soil samples used to spatialize soil properties, GSA ranked first in 25% of studies based on 10–100 samples per km^2^ (but there were few studies available) and in 23% of studies with densities of 0.1–1 sample per km^2^. However, in studies performed using < 0.1 samples per km^2^, PSA and HA provided the best performance in 56% and 42% of cases, respectively and only 2% for GSA (Fig 2D, S3 Table).

### The second group of studies: on the use frequency of soil mapping approaches

#### Spatialization approaches and techniques

Among the 33 different techniques used in the reviewed papers, 23 belonged to PSA, 2 belonged to GSA, and 8 belonged to HA (Fig 3). The most applied technique was Partial Least Squares Regression (PLSR; used in 22 studies). Random Forest (RF) was applied in 13 studies, followed by Multi Linear Regressions (MLR; 10 studies) and Ordinary Kriging (OK; 9 studies). Particular applications of kriging were also used in a few papers, such as block kriging, universal kriging, and kriging with external drift (all HA; Fig 3). Generally, almost 10% of the studies used GSA, 78% used PSA, and 12% used HA (S2 Table).

#### Soil properties

Concerning the categories of soil properties, organic carbon was the most considered (n = 52), followed by the grain size distribution (n = 29), whereas inorganic carbon was the least studied (n = 6; Fig 4B). PSA was used to map all types of soil properties and represents the most used approach across all categories of soil properties. GSA was used to map 9 of the 11 categories of soil properties and was used in 20–27% of the studies aimed at spatializing phosphorous, potassium, and other elements (Fig 4B). Finally, HA was used to spatialize only 6 soil categories, but was used in 57% of the studies aimed at spatializing phosphorus.

#### Density of observations

Studies using < 0.1 samples per km^2^ were the most frequent (37%), whereas studies using observation densities between 1.1 and 10 per km^2^ were the least frequent (7%; Fig 4C). PSA was the most used approach (75% of the studies) in all density categories. GSA was only used for low- and high-density classes. In contrast, HA has been scarcely applied regardless of the density category, except for studies using 10–100 samples per km^2^ (33% of them).

#### Extent

The highest percentage of studies (31%) concerned large study areas (> 10000 km^2^), whereas the lowest percentage (5%) applied to extents between 1.1 and 10 km^2^ (Fig 4D). Studies carried out in very large areas (> 10000 km^2^, i.e., the most frequent extents) used PSA in 87% of cases, and none of these studies mentioned HA. In study areas ranging from 1000 to 10000 km^2^, approximately 95% of studies used HA. A total of 55% of the study areas with extents ranging from 100 to 1000 km^2^ referred to PSA (Fig 3), 33% referred to HA and 22% to GSA (Fig 4D). In areas ranging from 1 to 1000 km^2^, PSA was used in more than 70% of cases, HA was used in 25% of the studies, whereas GSA was mostly used in studies of smaller extents (<1 km^2^), but these were weakly represented.

#### Altitudinal range

The highest percentage of studies, i.e. 32%, concerned areas with an altitudinal range between 100 and 500 m. In contrast, only 9% of them were conducted in areas with an altitudinal range between 1000 and 2000 m (Fig 4E). PSA approaches were used in all studies carried out in heterogeneous areas with an altitudinal variation between 1000 and 2000 m and in 85% of studies carried out in areas > 2000 m. In contrast, only 50% of the studies concerning nearly flat areas in the lowlands (between 1 and 100 m of altitudinal range) used PSA. In this category, GSA and HA were both used in 25% of the studies. HA was only used in another category, the 500–1000 m range, but in 40% of these studies. GSA was applied in four different altitudinal ranges and represented 20% to 25% of its use.

#### Continent of origin and year of publication

Europe had the highest percentage of studies (45%). Whereas PSA was used worldwide, almost 29% of Latin American studies used GSA, and none used HA (Fig 4F). The opposite situation was observed in Australia, where 30% of the studies used HA but none used GSA. The year with the highest number of studies overall was 2014 (24%). The year with the highest number of papers using HA was 2012 (almost 28%), whereas 2010 was the year with the highest percentage of studies using GSA. In 2011, all studies used PSA.

### Performance of approaches and their frequency of use

By examining comparative studies (i.e. using >1 approach; Fig 2) and statistics of the use of soil mapping approaches (Fig 4), we evidenced that GSA was the approach performing worst (Fig 2A) and, accordingly, tended to be decreasingly applied (Fig 4A). Hereafter, we consider only situations for which > 10 analyses were available in comparative studies.

Concerning the classes of soil properties, particularly grain-size distributions, organic carbon, and chemical properties, PSA was overly used (80%), compared with the best performance percentage (50–60%). It was the opposite for HA, which was underused (10–15%) compared to its best performance percentage. For chemical properties, GSA was never the best performing approach, and it was used in only 13% of the studies (Fig 2B and Fig 4B). For available data of soil density classes, PSA was overly used (85%) compared to its best performance percentage (56%); in contrast, HA was underused (56% of the best performance percentage compared to 12% of use) in studies carried out with a very low density of soil samples (< 0.1 per km^2^; Fig 2 and Fig 4).

Regarding classes of study area extent, the available data showed the same situation for areas ranging between 100 and 1000 km^2^, i.e. PSA being overly used compared to its best performance percentage, and HA being underused despite its greater performance. Regarding areas > 10000 km^2^, PSA was the best performing approach in 68% of cases and HA in 32% (Fig 2); however, looking at the percentage of use, PSA was applied in 90% of cases, GSA in 10%, and HA was never used (Fig 2, Fig 4 and S4 Table).

## Discussion

By analysing comparative studies and ordinary studies that mapped soil properties, our results suggest discrepancies between the performance of approaches and their frequency of use. In the literature, predictive statistical approaches (PSA) were more widely used than geostatistical (GSA) and hybrid approaches (HA; mixing PSA and GSA). However, comparison studies showed that PSA was not the best performing in all situations, being challenged by HA in several instances, although the latter was much less used in practice when authors had to choose a single approach.

Furthermore, and perhaps unsurprisingly, results from our review did not allow identifying any clear trends in the choice of the approach to use for mapping specific soil proprieties. Papers that compared the use of different approaches for a given soil property did not find trends in the evaluation values, resulting in a different approach performing best for a same property in different study contexts. For instance, in Guo et al. [18], the authors mapped soil organic matter (SOM) using HA and GSA, with many data available and densely distributed. They showed that random forest combined with residual kriging (HA) worked better than simple random forest (PSA). On the contrary, in Cambule et al. [30] the density of SOM data was low, HA based on kriging with external drift had the same performance than PSA based on linear regression, whereas GSA based on ordinary kriging gave the worst results. HA worked best when there was a large spatial correlation of the error between data, but was constrained by data availability, in which case GSA and HA methods did not work [30]. This data limitation effect could therefore not be identified in other studies (on different soil properties) where, for example, large datasets were available and densely distributed (e.g. [31–34]). The choice of a specific approach thus does not seem to be driven by a specific soil parameter, nor by characteristics of the study area or the number of soil samples, but rather by the researchers’ personal choices.

### Spatialization approaches

Across the 105 analysed papers, 37 different techniques belonging to one of the three main approaches (PSA, GSA and HA) were used to spatialize soil properties. Interestingly, PSA was used almost four times more than GSA or HA to map soil properties. Partial least squares regression (PLSR), random forest (RF), and multiple linear regression (MLR) were the three techniques most used, all belonging to PSA. Next were ordinary kriging (OK) and regression kriging (RK), belonging to GSA and HA respectively. Kriging techniques have a particularly long tradition in soil mapping [30, 35–38]. Burgess and Webster [39, 40] were the first to introduce OK to the soil community; since then, a large amount of studies using OK has been published. However, during the last 20 years, OK (and in general GSA) has been criticized as being excessively data-dependent, requiring a large number of regularly spaced data points, assuming significant trends in spatial autocorrelation [9]. This is sometimes considered as a poor assumption in complex terrains, where abrupt changes can occur over very short distances [9], as evidenced in several papers included in this review. Studies that compared OK with other techniques showed that it generally displayed lower validation values ([30, 35–38], Fig 2, S3 Table), and only a few studies showed the best performance for OK. In a study by Hoffmann et al. [29] in the Swiss Alps (at elevations between 900 and 2400 m), OK scored higher than HA techniques, such as block kriging and regression kriging. However, the best results for OK in this mountain study can be explained by the high density of available samples (more than 47 per km^2^) and the small extent of the study area (8.6 km^2^; [29]). OK has been modified in a variety of ways to incorporate ancillary data (e.g., soil-landscape relationships), turning it into a hybrid approach such as regression kriging, which turned out to be one of the most used among HA in this review. The latter combines a regression of the dependent variable on auxiliary (i.e., usually environmental) variables with kriging of the regression residuals. It is mathematically equivalent to the interpolation method variously called universal kriging, kriging with external drift, or co-kriging, where auxiliary environmental predictors are used directly to solve the kriging weights [8, 24]. These HA techniques were performing better than PSA and GSA in several selected papers comparing approaches (Fig 2, S3 Table), but they remained surprisingly infrequently used (Fig 4). Instead, PSA, which exploits the relationships between soil properties and environmental parameters to create predictive soil maps, has become the most used (Fig 4). Both HA and PSA need accurate digital maps of auxiliary environmental factors, which, in the past, were available for only small study areas [9]. For this reason, in the past, PSA and HA were less used, and a large proportion of existing studies were conducted in landscapes of small extents [9]. The great developments in computational power and geographic information technologies have resulted in a large increase in the amount of numeric geodata and geotechnology. In particular, the increasing power of tools such as geographic information systems (GIS), global positioning systems (GPS), remote and proximal sensor imagery (RSI), and associated data sources, such as very high-resolution digital elevation models (VHR DEMs), suggest new ways forward [41]. These advances allowed very detailed environmental data to be mapped across very large areas and thus PSA (and potentially HA) to be used across increasingly large extents. For instance, Poggio et al. [42] produced maps of soil organic matter for the whole surface of Scotland using predictive statistical approaches (GAM, RT) applied to ancillary data derived from remote sensing images.

### Modelled soil properties

For both groups of papers, the comparative ones and those that consider only one approach, Soil Organic Carbon (SOC) and grain-size distributions were the most modelled categories of soil properties. The other properties were much less frequently modelled. Grain-size distribution classes, including total coarse fragments, cobble gravels, sand, silt, and clay have been widely modelled at the field and farm scale because of their importance in agriculture. Proximal soil sensors are often proposed as technical solutions [43], with VIS-NIR (visible-near infra-red) spectroscopy being often used for soil texture mapping. Absorption in the VIS-NIR range of the electromagnetic spectrum can relate to soil properties because of absorption by molecules related to clay minerals.

SOC is recognised as the largest supply of terrestrial carbon [44]. Globally, the carbon storage capacity is considerably higher in terrestrial soils than in the atmosphere or in vegetation, making its mapping of growing interest, as proven by the increasing number of publications mapping SOC globally or countrywide [10]. In this review, we identified several studies spatializing organic carbon in mountainous environments (e.g., [29, 36, 45, 46]). The loss of SOC is also a global issue of increasing concern [47]. It can be particularly challenging in mountain environments, where mountain soils are characterized by a coarse texture and mostly rely on high organic carbon contents to resist erosion in harsh climatic conditions [47]. A decreased resistance to erosion and degradation of Alpine soils play an important role in the hydrogeological cycle of mountain environments, which is likely to result in an increasing frequency of natural disaster occurrences in these areas [48]. In such environments, 80% of the studies spatializing SOC and grain-size distributions used PSA. It is known that patterns of SOC and soil texture exhibit high spatial variations [41], and SOC studies require efficient and intense sampling strategies to reproduce the observed variations when using GSA, whereas PSA necessitates much less data for a similar outcome [29]. Regarding HA techniques, they outperformed PSA for only a few soil properties. This observation probably reflects the lack of theoretical knowledge regarding the processes behind the spatial variations of these properties [49], which is required to use HA (e.g. to fit a realistic model through a semi-variogram in kriging), instead of relying only on ‘aspatial’ soil-environment relationships in PSA. Thus, HA seems particularly useful when there is a large spatial correlation of the PSA model errors and when available environmental factors alone cannot explain the spatial distribution of the mapped soil properties.

### Characteristics of the study areas

In very large areas (1000–10000 km^2^), GSA has been used in only 11–12% of the studies. This may be explained by the increasing availability of global environmental datasets (e.g. worldclim, global DEM, PANGEA, EarthEnv, etc), allowing the use of PSA and HA at these scales. In very small areas, the predominant use of PSA can be explained by the availability of local environmental maps, whereas the most missing data are environmental maps at a medium resolution and regional scale. The majority of studies have been carried out using a low density of samples (< 1 per km^2^) and, in these cases, PSA has been the approach the most used. This is likely explained by the geographical variation between point locations that are too distant and not being reproducible by their geographic positions only. However, GSA has performed well in a few cases in complex landscapes characterized by wide elevation ranges, but these were usually studies within small extents with points sampled over small distances, where a sufficient complexity in the GSA approach was able to capture this variation. Thus, this is not in contradiction with the principle of Hoffmann et al. [29] that, if large variations exist over small distances, high quality GSA (*e*.*g*., kriging) will need a very large number of points (much higher than PSA or HA) to allow all of the terrain variation to be captured.

### Not all combinations of categories assessed

Finally, our bibliographic search showed that comparative studies do not exist for all combinations of the categories considered in this paper, with none or only very few studies being available for some situations, such as across different altitudinal ranges in very small study area extents or, oppositely, sufficient sample densities (> 1 sample per km^2^) across large extents. Several categories of soil properties have also been understudied, such as nitrogen, potassium, exchange bases and ions, and several other minor elements. The lack of some considered categories can probably be due to the restriction to three journals considered in our search. But this restriction to a limited number of journals, and a given period, was necessary to provide a clear framework to such a meta-analysis, to make it repeatable, and to allow objective trends to be identified. We found other reviews that also restricted their search to three highly-ranked international soil science journals during 10 years, for a total of 40 papers analysed [24]. In this regard, we encourage future reviews of soil digital mapping approaches to repeat the same type of meta-analyses across a much larger number of soil journals and possibly longer time period.

## Conclusions and some guidance

Although the comparative studies included in this review do not cover all types of situations, it still allows drawing some useful (but not definitive) conclusions:

1) GSA proved to be generally the worst performing and PSA the best performing approaches in the majority of cases, even if HA seems to increasingly challenge PSA in several domains, particularly when available environmental factors alone cannot explain the spatial distribution of certain soil properties, especially for large study area extents (>10000 km^2^) with a low density of samples (<1/km^2^). However, HA seems to work best when there is a large remaining spatial autocorrelation of the errors from a PSA model. The other limitation is related to the type of data available: if data were obtained through preferential or clustered sampling, then GSA and HA methods tend to fail. This study tends to confirm previous results, but with a significantly larger sample size. Yet, the most used and overall powerful approach—PSA—is outperformed by HA in several cases (and very rarely by GSA), especially when the distribution of PSA model errors can be geographically clustered. Certainly, one would have to read into the detail of the results and discussion of all the papers to see if authors at least tried HA or GSA, before reaching the conclusion that geostatistical modelling does not improve predictions when combined to PSA. Resuming the information from a larger number of individual papers would still be needed before reaching a definitive conclusion.

2) For the three classes of soil properties for which more data are available, i.e. grain-size distributions, organic carbon, and chemical properties, and for very large areas and for studies with very low densities of samples, the frequency with which a given spatialization approach was used did not systematically reflect its better performance overall. PSA was used in a larger number of cases than those in which it was the best performing, and HA was comparatively less used although often well performing. In this regard, our review suggests that HA could be used more often as an alternative to PSA when environmental factors alone are not able to capture the full spatial variation in soil properties, i.e. when errors from PSA models show spatial autocorrelation patterns.

3) In our review, comparative studies were not available for all combinations of situations, preventing recommendations to be drawn about the best performing approaches in some specific situations such as very small and intermediate study areas extent, very highly densely distributes sample points and for some soil properties categories (potassium, general descriptor and other elements). More studies are needed for these specific settings, which would help to establish more comprehensively which approach is the best fitted for what type of soil property, study area, and/or sampling density. Our review was such a first attempt, trying to help and guide selection of the most suitable digital soil modelling for different situations. Here, we mainly aimed at focusing on the difference between the three broad categories of approaches—PSA that uses the relationship between soil properties and environmental variables but does not use the spatial information, GSA that only uses the spatial information and HA that mixes both—but a more advanced and comprehensive review of studies that also more systematically considers single techniques belonging to the same DSM approach would still be needed in the future. Being able to identify the best technique within each category in a specified situation would then represent a great further advancement. However, as these are based on the same general principle characterizing each approach, any step done to help identifying the right category–as provided here–can be considered a useful achievement.

Moving forward more quantitatively with such assessment of the best approach and technique to use for digital soil mapping will require integrating field surveys and advanced numerical technologies in a multidisciplinary approach within the same geographic area, in such a way that one component can serve to predict another. This could lead to the development of a more integrated framework on which such methodological decisions can be based.

## Supporting information

S1 Appendix List of 373 papers resulted from the search. (DOCX)

S1 Table List of the 12 papers used in validation of comparison between the different method techniques. In bold are evidenced the best validation values. ANN = Artificial Neural Network; OK = Ordinary Kriging; UK = Universal Kriging; IDW = Inverse Distance Weighted; SLR = Stepwise Linear Regression; RF = Random Forest; RK = Regression Kriging; BK = Block Kriging; RST = Regularized Spline with Tension; OCK = Ordinary Co-Kriging; HASM = High accuracy surface modelling; HASM_LU = High Accuracy Surface Modelling with Land Use information; OK_LU = Ordinary Kriging with Land Use information; SK = Stratified Kriging; RK_GLM = Regression-Kriging using a Generalized Linear Model; RK/SMLR = regression kriging using stepwise multiple linear regression; RK/RT = regression kriging using regression trees to map the global spatial trend; DS = Disaggregation simulation; AWM = Area-weighted mean; KED = Kriging with external drift; KST = Kriging combined with Soil-Type information; KLU = Kriging combined with Land Use; KLUST = Kriging combined with Soil Type; RK(TOPO, TOPOVI, TOPOSOIL and TOPOVISOIL) = Regression Kriging with four auxiliary data sets; GWR = Geographically Weighted Regression. CKmc = multi-collocated CoKriging. RMSE = Root Mean Square Error; Var = Variance explained (%); R^2^ = Coefficient of determination; MAE = Mean Absolute Error; RPD = Residual Prediction.
(DOCX)

S2 Table List of the 92 selected papers using only one type of approach to spatialize soil properties. (DOCX)

S3 Table The first group: comparative studies. Total number of studies for a. each type of approach (GSA: geostatistical approaches, PSA: predictive statistical approaches, HA: hybrid approaches); b. soil properties category and percentage of analyses used in each approach that obtained the highest spatialization performance value; c. study area extent category and percentage of analyses used in each approach that obtained the highest spatialization performance value; d. sample density category and percentage of analyses used in each approach that obtained the highest spatialization performance value.
(DOCX)

S4 Table The second group of studies: On the use frequency of soil mapping approaches. Total number of studies for: a. each type of approach (GSA: geostatistical approaches, PSA: predictive statistical approaches, HA: hybrid approaches); b. each soil properties category and percentage of analyses used in each approach; c. each study area extent category and percentage of analyses used in each approach; d. each sample density category and percentage of analyses used in each approach; e. each altitudinal range category and percentage of analyses used in each approach; f. each year considered in the review and percentage of analyses used in each approach; g. continent and percentage of analyses used in each approach.
(DOCX)
