# Supplementary material for: Generalizing soil properties in geographic space: Approaches used and ways forward
Source: PLoS One. 2018 Dec 21;13(12):e0208823. doi: 10.1371/journal.pone.0208823 (PMC6303050; doi:10.1371/journal.pone.0208823)
Supplement: S1 Appendix — (DOCX) [file pone.0208823.s001.docx]

**S1 Appendix - List of 373 papers resulted from the search**

Abu, S.T. & Abubakar, I.U. (2013) Evaluating the effects of tillage techniques on soil hydro-physical properties in Guinea Savanna of Nigeria. *Soil & Tillage Research,* **126,** 159-168.

Adhikari, K. & Hartemink, A.E. (2016) Linking soils to ecosystem services - A global review. *Geoderma,* **262,** 101-111.

Aiello, R., Bagarello, V., Barbagallo, S., Consoli, S., Di Prima, S., Giordano, G. & Iovino, M. (2014) An assessment of the Beerkan method for determining the hydraulic properties of a sandy loam soil. *Geoderma,* **235,** 300-307.

Aitkenhead, M.J., Coull, M., Towers, W., Hudson, G. & Black, H.I.J. (2013) Prediction of soil characteristics and colour using data from the National Soils Inventory of Scotland. *Geoderma,* **200,** 99-107.

Aldana-Jague, E., Heckrath, G., Macdonald, A., van Wesemael, B. & Van Oost, K. (2016) UAS-based soil carbon mapping using VIS-NIR (480-1000 nm) multi-spectral imaging: Potential and limitations. *Geoderma,* **275,** 55-66.

Algayer, B., Wang, B., Bourennane, H., Zheng, F., Duval, O., Li, G., Le Bissonnais, Y. & Darboux, F. (2014) Aggregate stability of a crusted soil: differences between crust and sub-crust material, and consequences for interrill erodibility assessment. An example from the Loess Plateau of China. *European Journal of Soil Science,* **65,** 325-335.

Altdorff, D., Bechtold, M., van der Kruk, J., Vereecken, H. & Huisman, J.A. (2016) Mapping peat layer properties with multi-coil offset electromagnetic induction and laser scanning elevation data. *Geoderma,* **261,** 178-189.

Amirinejad, A.A., Kamble, K., Aggarwal, P., Chakraborty, D., Pradhan, S. & Mittal, R.B. (2011) Assessment and mapping of spatial variation of soil physical health in a farm. *Geoderma,* **160,** 292-303.

Angelini, M.E., Heuvelink, G.B.M., Kempen, B. & Morras, H.J.M. (2016) Mapping the soils of an Argentine Pampas region using structural equation modelling. *Geoderma,* **281,** 102-118.

Antinoro, C., Bagarello, V., Ferro, V., Giordano, G. & Iovino, M. (2014) A simplified approach to estimate water retention for Sicilian soils by the Arya-Paris model. *Geoderma,* **213,** 226-234.

Aquino, L.S., Timm, L.C., Reichardt, K., Barbosa, E.P., Parfitt, J.M.B., Nebel, A.L.C. & Penning, L.H. (2015) State-space approach to evaluate effects of land levelling on the spatial relationships of soil properties of a lowland area. *Soil & Tillage Research,* **145,** 135-147.

Araujo, S.R., Soderstrom, M., Eriksson, J., Isendahl, C., Stenborg, P. & Dematte, J.A.M. (2015) Determining soil properties in Amazonian Dark Earths by reflectance spectroscopy. *Geoderma,* **237,** 308-317.

Araujo, S.R., Wetterlind, J., Dematte, J.A.M. & Stenberg, B. (2014) Improving the prediction performance of a large tropical vis-NIR spectroscopic soil library from Brazil by clustering into smaller subsets or use of data mining calibration techniques. *European Journal of Soil Science,* **65,** 718-729.

Arkhangelskaya, T.A. (2014) Diversity of thermal conditions within the paleocryogenic soil complexes of the East European Plain: The discussion of key factors and mathematical modeling. *Geoderma,* **213,** 608-616.

Arnhold, S., Otieno, D., Onyango, J., Koellner, T., Huwe, B. & Tenhunen, J. (2015) Soil properties along a gradient from hillslopes to the savanna plains in the Lambwe Valley, Kenya. *Soil & Tillage Research,* **154,** 75-83.

Arvidsson, J., Westlin, H., Keller, T. & Gilbertsson, M. (2011) Rubber track systems for conventional tractors - Effects on soil compaction and traction. *Soil & Tillage Research,* **117,** 103-109.

Askari, M.S., Cui, J.F., O'Rourke, S.M. & Holden, N.M. (2015) Evaluation of soil structural quality using VIS-NIR spectra. *Soil & Tillage Research,* **146,** 108-117.

Bagarello, V., Di Prima, S., Giordano, G. & Iovino, M. (2014) A test of the Beerkan Estimation of Soil Transfer parameters (BEST) procedure. *Geoderma,* **221,** 20-27.

Ballabio, C., Fava, F. & Rosenmund, A. (2012) A plant ecology approach to digital soil mapping, improving the prediction of soil organic carbon content in alpine grasslands. *Geoderma,* **187,** 102-116.

Ballabio, C., Panagos, P. & Monatanarella, L. (2016) Mapping topsoil physical properties at European scale using the LUCAS database. *Geoderma,* **261,** 110-123.

Batjes, N.H. (2016) Harmonized soil property values for broad-scale modelling (WISE30sec) with estimates of global soil carbon stocks. *Geoderma,* **269,** 61-68.

Bayat, H., Neyshaburi, M.R., Mohammadi, K., Nariman-Zadeh, N. & Irannejad, M. (2013) Improving water content estimations using penetration resistance and principal component analysis. *Soil & Tillage Research,* **129,** 83-92.

Behera, S.K., Singh, M.V., Singh, K.N. & Todwal, S. (2011) Distribution variability of total and extractable zinc in cultivated acid soils of India and their relationship with some selected soil properties. *Geoderma,* **162,** 242-250.

Behrens, T., Schmidt, K., Ramirez-Lopez, L., Gallant, J., Zhu, A.X. & Scholten, T. (2014) Hyper-scale digital soil mapping and soil formation analysis. *Geoderma,* **213,** 578-588.

Beucher, A., Siemssen, R., Frojdo, S., Osterholm, P., Martinkauppi, A. & Eden, P. (2015) Artificial neural network for mapping and characterization of acid sulfate soils: Application to Sirppujoki River catchment, southwestern Finland. *Geoderma,* **247,** 38-50.

Bevington, J., Piragnolo, D., Teatini, P., Vellidis, G. & Morari, F. (2016) On the spatial variability of soil hydraulic properties in a Holocene coastal farmland. *Geoderma,* **262,** 294-305.

Bird, N.R.A. & Perrier, E. (2010) Multiscale percolation properties of a fractal pore network. *Geoderma,* **160,** 105-110.

Biswas, A., Cresswell, H.P., Rossel, R.A.V. & Si, B.C. (2013) Characterizing scale- and location-specific variation in non-linear soil systems using the wavelet transform. *European Journal of Soil Science,* **64,** 706-715.

Biswas, A., Cresswell, H.P., Rossel, R.A.V. & Si, B.C. (2014) Curve let transform to study scale-dependent anisotropic soil spatial variation. *Geoderma,* **213,** 589-599.

Bitencourt, D.G.B., Barros, W.S., Timm, L.C., She, D.L., Penning, L.H., Parfitt, J.M.B. & Reichardt, K. (2016) Multivariate and geostatistical analyses to evaluate lowland soil levelling effects on physico-chemical properties. *Soil & Tillage Research,* **156,** 63-73.

Bockheim, J.G. & Gennadiyev, A.N. (2015) General state soil maps in the USA. *Geoderma,* **253,** 78-89.

Bogunovic, I., Mesic, M., Zgorelec, Z., Jurisic, A. & Bilandzija, D. (2014) Spatial variation of soil nutrients on sandy-loam soil. *Soil & Tillage Research,* **144,** 174-183.

Bonilla, C.A. & Johnson, O.I. (2012) Soil erodibility mapping and its correlation with soil properties in Central Chile. *Geoderma,* **189,** 116-123.

Bossa, A.Y., Diekkruger, B., Igoe, A.M. & Gaiser, T. (2012) Analyzing the effects of different soil databases on modeling of hydrological processes and sediment yield in Benin (West Africa). *Geoderma,* **173,** 61-74.

Boudreault, J.P., Dube, J.S. & Marcotte, D. (2016) Quantification and minimization of uncertainty by geostatistical simulations during the characterization of contaminated sites: 3-D approach to a multi-element contamination. *Geoderma,* **264,** 214-226.

Bragato, G., Fornasier, F. & Brus, D.J. (2016) Characterization of soil fertility and soil biodiversity with dsDNA as a covariate in a regression estimator for mean microbial biomass C. *European Journal of Soil Science,* **67,** 827-834.

Brevik, E.C., Calzolari, C., Miller, B.A., Pereira, P., Kabala, C., Baumgarten, A. & Jordan, A. (2016) Soil mapping, classification, and pedologic modeling: History and future directions. *Geoderma,* **264,** 256-274.

Brus, D.J. (2015) Balanced sampling: A versatile sampling approach for statistical soil surveys. *Geoderma,* **253,** 111-121.

Bullinger-Weber, G., Le Bayon, R.C., Thebault, A., Schlaepfer, R. & Guenat, C. (2014) Carbon storage and soil organic matter stabilisation in near-natural, restored and embanked Swiss floodplains. *Geoderma,* **228,** 122-131.

Burak, D.L., Fontes, M.P.F., Santos, N.T., Monteiro, L.V.S., Martins, E.D. & Becquer, T. (2010) Geochemistry and spatial distribution of heavy metals in Oxisols in a mineralized region of the Brazilian Central Plateau. *Geoderma,* **160,** 131-142.

Calzolari, C., Ungaro, F., Filippi, N., Guermandi, M., Malucelli, F., Marchi, N., Staffilani, F. & Tarocco, P. (2016) A methodological framework to assess the multiple contributions of soils to ecosystem services delivery at regional scale. *Geoderma,* **261,** 190-203.

Camargo, L.A., Marques, J., Barron, V., Alleoni, L.R.F., Barbosa, R.S. & Pereira, G.T. (2015) Mapping of clay, iron oxide and adsorbed phosphate in Oxisols using diffuse reflectance spectroscopy. *Geoderma,* **251,** 124-132.

Cambule, A.H., Rossiter, D.G., Stoorvogel, J.J. & Smaling, E.M.A. (2012) Building a near infrared spectral library for soil organic carbon estimation in the Limpopo National Park, Mozambique. *Geoderma,* **183,** 41-48.

Canasveras, J.C., Barron, V., del Campillo, M.C., Torrent, J. & Gomez, J.A. (2010) Estimation of aggregate stability indices in Mediterranean soils by diffuse reflectance spectroscopy. *Geoderma,* **158,** 78-84.

Caron, E., Farenhorst, A., Zvomuya, F., Gaultier, J., Rank, N., Goddard, T. & Sheedy, C. (2010) Sorption of four estrogens by surface soils from 41 cultivated fields in Alberta, Canada. *Geoderma,* **155,** 19-30.

Casa, R., Castaldi, F., Pascucci, S., Palombo, A. & Pignatti, S. (2013) A comparison of sensor resolution and calibration strategies for soil texture estimation from hyperspectral remote sensing. *Geoderma,* **197,** 17-26.

Castaldi, F., Casa, R., Castrignano, A., Pascucci, S., Palombo, A. & Pignatti, S. (2014) Estimation of soil properties at the field scale from satellite data: a comparison between spatial and non-spatial techniques. *European Journal of Soil Science,* **65,** 842-851.

Chakraborty, S., Weindorf, D.C., Zhu, Y.D., Li, B., Morgan, C.L.S., Ge, Y.F. & Galbraith, J. (2012) Spectral reflectance variability from soil physicochemical properties in oil contaminated soils. *Geoderma,* **177,** 80-89.

Chandrasoma, J.M., Udawatta, R.P., Anderson, S.H., Thompson, A.L. & Abney, M.A. (2016) Soil hydraulic properties as influenced by prairie restoration. *Geoderma,* **283,** 48-56.

Chaplot, V., Lorentz, S., Podwojewski, P. & Jewitt, G. (2010) Digital mapping of A-horizon thickness using the correlation between various soil properties and soil apparent electrical resistivity. *Geoderma,* **157,** 154-164.

Chirico, G.B., Medina, H. & Romano, N. (2010) Functional evaluation of PTF prediction uncertainty: An application at hillslope scale. *Geoderma,* **155,** 193-202.

Ciampalini, R., Lagacherie, P., Gomez, C., Grunberger, O., Hamrouni, M.H., Mekki, I. & Richard, A. (2013) Detecting, correcting and interpreting the biases of measured soil profile data: A case study in the Cap Bon Region (Tunisia). *Geoderma,* **192,** 68-76.

Cihlar, Z., Vojtova, L., Michlovska, L. & Kucerik, J. (2016) Preparation and hydration characteristics of carbodiimide crosslinked lignite humic acids. *Geoderma,* **274,** 10-17.

Clifford, D., Dobbie, M.J. & Searle, R. (2014) Non-parametric imputation of properties for soil profiles with sparse observations. *Geoderma,* **232,** 10-18.

Cobo, J.G., Dercon, G., Yekeye, T., Chapungu, L., Kadzere, C., Murwira, A., Delve, R. & Cadisch, G. (2010) Integration of mid-infrared spectroscopy and geostatistics in the assessment of soil spatial variability at landscape level. *Geoderma,* **158,** 398-411.

Colombo, C., Palumbo, G., Sellitto, V.M., Di Iorio, E., Castrignano, A. & Stelluti, M. (2015) The effects of land use and landscape on soil nitrate availability in Southern Italy (Molise region). *Geoderma,* **239,** 1-12.

Coppola, A., Comegna, A., Dragonetti, G., Lamaddalena, N., Kader, A.M. & Comegna, V. (2011) Average moisture saturation effects on temporal stability of soil water spatial distribution at field scale. *Soil & Tillage Research,* **114,** 155-164.

Coppola, A., Dragonetti, G., Comegna, A., Lamaddalena, N., Caushi, B., Haikal, M.A. & Basile, A. (2013) Measuring and modeling water content in stony soils. *Soil & Tillage Research,* **128,** 9-22.

Cornu, J.Y., Schneider, A., Jezequel, K. & Denaix, L. (2011) Modelling the complexation of Cd in soil solution at different temperatures using the UV-absorbance of dissolved organic matter. *Geoderma,* **162,** 65-70.

Corwin, D.L. & Lesch, S.M. (2014) A simplified regional-scale electromagnetic induction - Salinity calibration model using ANOCOVA modeling techniques. *Geoderma,* **230,** 288-295.

Coulouma, G., Caner, L., Loonstra, E.H. & Lagacherie, P. (2016) Analysing the proximal gamma radiometry in contrasting Mediterranean landscapes: Towards a regional prediction of clay content. *Geoderma,* **266,** 127-135.

Coulouma, G., Samyn, K., Grandjean, G., Follain, S. & Lagacherie, P. (2012) Combining seismic and electric methods for predicting bedrock depth along a Mediterranean soil toposequence. *Geoderma,* **170,** 39-47.

Cruz-Cardenas, G., Lopez-Mata, L., Ortiz-Solorio, C.A., Villasenor, J.L., Ortiz, E., Silva, J.T. & Estrada-Godoy, F. (2014) Interpolation of Mexican soil properties at a scale of 1:1,000,000. *Geoderma,* **213,** 29-35.

Czobel, S., Horvath, L., Szirmai, O., Balogh, J., Pinter, K., Nemeth, Z., Urmos, Z., Grosz, B. & Tuba, Z. (2010) Comparison of N2O and CH4 fluxes from Pannonian natural ecosystems. *European Journal of Soil Science,* **61,** 671-682.

D'Acqui, L.P., Pucci, A. & Janik, L.J. (2010) Soil properties prediction of western Mediterranean islands with similar climatic environments by means of mid-infrared diffuse reflectance spectroscopy. *European Journal of Soil Science,* **61,** 865-876.

D'Or, D. & Destain, M.F. (2014) Toward a tool aimed to quantify soil compaction risks at a regional scale: Application to Wallonia (Belgium). *Soil & Tillage Research,* **144,** 53-71.

da Silva, A.F., Pereira, M.J., Cameiro, J.D., Zimback, C.R.L., Landim, P.M.B. & Soares, A. (2014) A new approach to soil classification mapping based on the spatial distribution of soil properties. *Geoderma,* **219,** 106-116.

Dabach, S., Shani, U. & Lazarovitch, N. (2016) The influence of water uptake on matric head variability in a drip-irrigated root zone. *Soil & Tillage Research,* **155,** 216-224.

Daryanto, S., Eldridge, D.J. & Wang, L.X. (2013) Ploughing and grazing alter the spatial patterning of surface soils in a shrub-encroached woodland. *Geoderma,* **200,** 67-76.

De Benedetto, D., Castrignano, A., Sollitto, D., Modugno, F., Buttafuoco, G. & lo Papa, G. (2012) Integrating geophysical and geostatistical techniques to map the spatial variation of clay. *Geoderma,* **171,** 53-63.

de Carvalho, W., Lagacherie, P., Chagas, C.D., Calderano, B. & Bhering, S.B. (2014) A regional-scale assessment of digital mapping of soil attributes in a tropical hillslope environment. *Geoderma,* **232,** 479-486.

Debaene, G., Niedzwiecki, J., Pecio, A. & Zurek, A. (2014) Effect of the number of calibration samples on the prediction of several soil properties at the farm-scale. *Geoderma,* **214,** 114-125.

Dessureault-Rompre, J., Zebarth, B.J., Georgallas, A., Burton, D.L. & Grant, C.A. (2011) A biophysical water function to predict the response of soil nitrogen mineralization to soil water content. *Geoderma,* **167-68,** 214-227.

Diek, S., Temme, A. & Teuling, A.J. (2014) The effect of spatial soil variation on the hydrology of a semi-arid Rocky Mountains catchment. *Geoderma,* **235,** 113-126.

Dierke, C. & Werban, U. (2013) Relationships between gamma-ray data and soil properties at an agricultural test site. *Geoderma,* **199,** 90-98.

Dietze, M. & Kleber, A. (2010) Characterisation and prediction of thickness and material properties of periglacial cover beds, Tharandter Wald, Germany. *Geoderma,* **156,** 346-356.

Doolittle, J.A. & Brevik, E.C. (2014) The use of electromagnetic induction techniques in soils studies. *Geoderma,* **223,** 33-45.

Dou, F.G., Yu, X., Ping, C.L., Michaelson, G., Guo, L.D. & Jorgenson, T. (2010) Spatial variation of tundra soil organic carbon along the coastline of northern Alaska. *Geoderma,* **154,** 328-335.

Farlin, J., Galle, T., Bayerle, M., Pittois, D., Braun, C., El Khabbaz, H., Lallement, C., Leopold, U., Vanderborght, J. & Weihermueller, L. (2013) Using the long-term memory effect of pesticide and metabolite soil residues to estimate field degradation half-life and test leaching predictions. *Geoderma,* **207,** 15-24.

Fatehnia, M., Tawfiq, K. & Ye, M. (2016) Estimation of saturated hydraulic conductivity from double-ring infiltrometer measurements. *European Journal of Soil Science,* **67,** 135-147.

Fromin, N., Saby, N.P.A., Lensi, R., Brunet, D., Porte, B., Domenach, A.M. & Roggy, J.C. (2013) Spatial variability of soil microbial functioning in a tropical rainforest of French Guiana using nested sampling. *Geoderma,* **197,** 98-107.

Fu, W.J., Tunney, H. & Zhang, C.S. (2010) Spatial variation of soil nutrients in a dairy farm and its implications for site-specific fertilizer application. *Soil & Tillage Research,* **106,** 185-193.

Gandois, L., Probst, A. & Dumat, C. (2010) Modelling trace metal extractability and solubility in French forest soils by using soil properties. *European Journal of Soil Science,* **61,** 271-286.

Gao, F., Feng, G., Sharratt, B. & Zhang, M. (2014) Tillage and straw management affect PM10 emission potential in subarctic Alaska. *Soil & Tillage Research,* **144,** 1-7.

Gao, W., Watts, C.W., Ren, T. & Whalley, W.R. (2012) The effects of compaction and soil drying on penetrometer resistance. *Soil & Tillage Research,* **125,** 14-22.

Garcia-Estringana, P., Alonso-Blazquez, N., Marques, M.J., Bienes, R. & Alegre, J. (2010) Direct and indirect effects of Mediterranean vegetation on runoff and soil loss. *European Journal of Soil Science,* **61,** 174-185.

Gasch, C.K., Huzurbazar, S.V. & Stahl, P.D. (2015) Small-scale spatial heterogeneity of soil properties in undisturbed and reclaimed sagebrush steppe. *Soil & Tillage Research,* **153,** 42-47.

Gasch, C.K., Huzurbazar, S.V. & Stahl, P.D. (2016) Description of vegetation and soil properties in sagebrush steppe following pipeline burial, reclamation, and recovery time. *Geoderma,* **265,** 19-26.

Ge, Y.F., Morgan, C.L.S. & Ackerson, J.P. (2014) VisNIR spectra of dried ground soils predict properties of soils scanned moist and intact. *Geoderma,* **221,** 61-69.

Glendining, M.J., Dailey, A.G., Powlson, D.S., Richter, G.M., Catt, J.A. & Whitmore, A.P. (2011) Pedotransfer functions for estimating total soil nitrogen up to the global scale. *European Journal of Soil Science,* **62,** 13-22.

Gobrecht, A., Bendoula, R., Roger, J.M. & Bellon-Maurel, V. (2016) A new optical method coupling light polarization and Vis-NIR spectroscopy to improve the measurement of soil carbon content. *Soil & Tillage Research,* **155,** 461-470.

Goge, F., Gomez, C., Jolivet, C. & Joffre, R. (2014) Which strategy is best to predict soil properties of a local site from a national Vis-NIR database? *Geoderma,* **213,** 1-9.

Gomez, C., Gholizadeh, A., Boruvka, L. & Lagacherie, P. (2016) Using legacy data for correction of soil surface clay content predicted from VNIR/SWIR hyperspectral airborne images. *Geoderma,* **276,** 84-92.

Gomez, C., Lagacherie, P. & Coulouma, G. (2012) Regional predictions of eight common soil properties and their spatial structures from hyperspectral Vis-NIR data. *Geoderma,* **189,** 176-185.

Gomez, C., Le Bissonnais, Y., Annabi, M., Bahri, H. & Raclot, D. (2013) Laboratory Vis-NIR spectroscopy as an alternative method for estimating the soil aggregate stability indexes of Mediterranean soils. *Geoderma,* **209,** 86-97.

Goovaerts, P. (2011) A coherent geostatistical approach for combining choropleth map and field data in the spatial interpolation of soil properties. *European Journal of Soil Science,* **62,** 371-380.

Graf, A., Herbst, M., Weihermuller, L., Huisman, J.A., Prolingheuer, N., Bornemann, L. & Vereecken, H. (2012) Analyzing spatiotemporal variability of heterotrophic soil respiration at the field scale using orthogonal functions. *Geoderma,* **181,** 91-101.

Gras, J.P., Barthes, B.G., Mahaut, B. & Trupin, S. (2014) Best practices for obtaining and processing field visible and near infrared (VNIR) spectra of topsoils. *Geoderma,* **214,** 126-134.

Grimm, R. & Behrens, T. (2010) Uncertainty analysis of sample locations within digital soil mapping approaches. *Geoderma,* **155,** 154-163.

Guadagnini, A., Neuman, S.P., Schaap, M.G. & Riva, M. (2014) Anisotropic statistical scaling of soil and sediment texture in a stratified deep vadose zone near Maricopa, Arizona. *Geoderma,* **214,** 217-227.

Guerrero, C., Zornoza, R., Gomez, I. & Mataix-Beneyto, J. (2010) Spiking of NIR regional models using samples from target sites: Effect of model size on prediction accuracy. *Geoderma,* **158,** 66-77.

Gwenzi, W., Hinz, C., Holmes, K., Phillips, I.R. & Mullins, I.J. (2011) Field-scale spatial variability of saturated hydraulic conductivity on a recently constructed artificial ecosystem. *Geoderma,* **166,** 43-56.

Hadzick, Z.Z., Guber, A.K., Pachepsky, Y.A. & Hill, R.L. (2011) Pedotransfer functions in soil electrical resistivity estimation. *Geoderma,* **164,** 195-202.

Hartemink, A.E., Krasilnikov, P. & Bockheim, J.G. (2013) Soil maps of the world. *Geoderma,* **207,** 256-267.

Haskard, K.A. & Lark, R.M. (2010) Spectral tempering to model non-stationary variation of soil properties: Sensitivity to the initial stationary model. *Geoderma,* **159,** 350-357.

Havaee, S., Mosaddeghi, M.R. & Ayoubi, S. (2015) In situ surface shear strength as affected by soil characteristics and land use in calcareous soils of central Iran. *Geoderma,* **237,** 137-148.

Hedley, C.B., Roudier, P., Yule, I.J., Ekanayake, J. & Bradbury, S. (2013) Soil water status and water table depth modelling using electromagnetic surveys for precision irrigation scheduling. *Geoderma,* **199,** 22-29.

Helliwell, J.R., Sturrock, C.J., Grayling, K.M., Tracy, S.R., Flavel, R.J., Young, I.M., Whalley, W.R. & Mooney, S.J. (2013) Applications of X-ray computed tomography for examining biophysical interactions and structural development in soil systems: a review. *European Journal of Soil Science,* **64,** 279-297.

Hemmat, A., Nankali, N. & Aghilinategh, N. (2012) Simulating stress-sinkage under a plate sinkage test using a viscoelastic 2D axisymmetric finite element soil model. *Soil & Tillage Research,* **118,** 107-116.

Herbst, M., Prolingheuer, N., Graf, A., Huisman, J.A., Weihermuller, L., Vanderborght, J. & Vereecken, H. (2010) Multivariate conditional stochastic simulation of soil heterotrophic respiration at plot scale. *Geoderma,* **160,** 74-82.

Herbst, M., Tappe, W., Kummer, S. & Vereecken, H. (2016) The impact of sieving on heterotrophic respiration response to water content in loamy and sandy topsoils. *Geoderma,* **272,** 73-82.

Heuvelink, G.B.M., Burgers, S., Tiktak, A. & Van Den Berg, F. (2010) Uncertainty and stochastic sensitivity analysis of the GeoPEARL pesticide leaching model. *Geoderma,* **155,** 186-192.

Hoffmann, U., Hoffmann, T., Jurasinski, G., Glatzel, S. & Kuhn, N.J. (2014) Assessing the spatial variability of soil organic carbon stocks in an alpine setting (Grindelwald, Swiss Alps). *Geoderma,* **232,** 270-283.

Hollis, J.M., Hannam, J. & Bellamy, P.H. (2012) Empirically-derived pedotransfer functions for predicting bulk density in European soils. *European Journal of Soil Science,* **63,** 96-109.

Hollis, J.M., Lilly, A., Higgins, A., Jones, R.J.A., Keay, C.A. & Bellamy, P. (2015) Predicting the water retention characteristics of UK mineral soils. *European Journal of Soil Science,* **66,** 239-252.

Horta, A. & Soares, A. (2010) Data integration model to assess soil organic carbon availability. *Geoderma,* **160,** 225-235.

Hu, K.L., Wang, S.Y., Li, H., Huang, F. & Li, B.G. (2014a) Spatial scaling effects on variability of soil organic matter and total nitrogen in suburban Beijing. *Geoderma,* **226,** 54-63.

Hu, W., Shao, M.A., Wan, L. & Si, B.C. (2014b) Spatial variability of soil electrical conductivity in a small watershed on the Loess Plateau of China. *Geoderma,* **230,** 212-220.

Hu, W. & Si, B.C. (2013) Soil water prediction based on its scale-specific control using multivariate empirical mode decomposition. *Geoderma,* **193,** 180-188.

Huang, H.B., Ouyang, W., Guo, B.B., Shi, Y.D. & Hao, F.H. (2014a) Vertical and horizontal distribution of soil parameters in intensive agricultural zone and effect on diffuse nitrogen pollution. *Soil & Tillage Research,* **144,** 32-40.

Huang, J., Lark, R.M., Robinson, D.A., Lebron, I., Keith, A.M., Rawlins, B., Tye, A., Kuras, O., Raines, M. & Triantafilis, J. (2014b) Scope to predict soil properties at within-field scale from small samples using proximally sensed gamma-ray spectrometer and EM induction data. *Geoderma,* **232,** 69-80.

Huang, M.B., Zettl, J.D., Barbour, S.L. & Pratt, D. (2016) Characterizing the spatial variability of the hydraulic conductivity of reclamation soils using air permeability. *Geoderma,* **262,** 285-293.

Hughes, P.A., McBratney, A.B., Minasny, B. & Campbell, S. (2014) End members, end points and extragrades in numerical soil classification. *Geoderma,* **226,** 365-375.

Hunt, A.G., Ghanbarian, B. & Saville, K.C. (2013) Unsaturated hydraulic conductivity modeling for porous media with two fractal regimes. *Geoderma,* **207,** 268-278.

Ingelmo, F., Molina, M.J., de Paz, J.M. & Visconti, F. (2011) Soil saturated hydraulic conductivity assessment from expert evaluation of field characteristics using an ordered logistic regression model. *Soil & Tillage Research,* **115,** 27-38.

Iwashita, F., Friedel, M.J., Ribeiro, G.F. & Fraser, S.J. (2012) Intelligent estimation of spatially distributed soil physical properties. *Geoderma,* **170,** 1-10.

Ji, W., Rossel, R.A.V. & Shi, Z. (2015a) Accounting for the effects of water and the environment on proximally sensed vis-NIR soil spectra and their calibrations. *European Journal of Soil Science,* **66,** 555-565.

Ji, W., Rossel, R.A.V. & Shi, Z. (2015b) Improved estimates of organic carbon using proximally sensed vis-NIR spectra corrected by piecewise direct standardization. *European Journal of Soil Science,* **66,** 670-678.

Ji, W.J., Li, S., Chen, S.C., Shi, Z., Rossel, R.A.V. & Mouazen, A.M. (2016) Prediction of soil attributes using the Chinese soil spectral library and standardized spectra recorded at field conditions. *Soil & Tillage Research,* **155,** 492-500.

Jia, X.X., Shao, M.G., Wei, X.R., Horton, R. & Li, X.Z. (2011) Estimating total net primary productivity of managed grasslands by a state-space modeling approach in a small catchment on the Loess Plateau, China. *Geoderma,* **160,** 281-291.

Jonard, F., Mahmoudzadeh, M., Roisin, C., Weihermuller, L., Andre, F., Minet, J., Vereecken, H. & Lambot, S. (2013) Characterization of tillage effects on the spatial variation of soil properties using ground-penetrating radar and electromagnetic induction. *Geoderma,* **207,** 310-322.

Jorda, H., Bechtold, M., Jarvis, N. & Koestel, J. (2015) Using boosted regression trees to explore key factors controlling saturated and near-saturated hydraulic conductivity. *European Journal of Soil Science,* **66,** 744-756.

Karamesouti, M., Petropoulos, G.P., Papanikolaou, I.D., Kairis, O. & Kosmas, K. (2016) Erosion rate predictions from PESERA and RUSLE at a Mediterranean site before and after a wildfire: Comparison & implications. *Geoderma,* **261,** 44-58.

Keller, T., Lamande, M., Peth, S., Berli, M., Delenne, J.Y., Baumgarten, W., Rabbel, W., Radjai, F., Rajchenbach, J., Selvadurai, A.P.S. & Or, D. (2013) An interdisciplinary approach towards improved understanding of soil deformation during compaction. *Soil & Tillage Research,* **128,** 61-80.

Kempen, B., Brus, D.J. & de Vries, F. (2015) Operationalizing digital soil mapping for nationwide updating of the 1:50,000 soil map of the Netherlands. *Geoderma,* **241,** 313-329.

Kempen, B., Brus, D.J. & Stoorvogel, J.J. (2011) Three-dimensional mapping of soil organic matter content using soil type-specific depth functions. *Geoderma,* **162,** 107-123.

Kempen, B., Heuvelink, G.B.M., Brus, D.J. & Stoorvogel, J.J. (2010) Pedometric mapping of soil organic matter using a soil map with quantified uncertainty. *European Journal of Soil Science,* **61,** 333-347.

Kerry, R., Goovaerts, P., Rawlins, B.G. & Marchant, B.P. (2012) Disaggregation of legacy soil data using area to point kriging for mapping soil organic carbon at the regional scale. *Geoderma,* **170,** 347-358.

Khlosi, M., Alhamdoosh, M., Douaik, A., Gabriels, D. & Cornelis, W.M. (2016) Enhanced pedotransfer functions with support vector machines to predict water retention of calcareous soil. *European Journal of Soil Science,* **67,** 276-284.

Kim, D. & Zheng, Y.B. (2011) Scale-dependent predictability of DEM-based landform attributes for soil spatial variability in a coastal dune system. *Geoderma,* **164,** 181-194.

Kim, I., Pullanagari, R.R., Deurer, M., Singh, R., Huh, K.Y. & Clothier, B.E. (2014) The use of visible and near-infrared spectroscopy for the analysis of soil water repellency. *European Journal of Soil Science,* **65,** 360-368.

Kinoshita, R., Roupsard, O., Chevallier, T., Albrecht, A., Taugourdeau, S., Ahmed, Z. & van Es, H.M. (2016) Large topsoil organic carbon variability is controlled by Andisol properties and effectively assessed by VNIR spectroscopy in a coffee agroforestry system of Costa Rica. *Geoderma,* **262,** 254-265.

Kodaira, M. & Shibusawa, S. (2013) Using a mobile real-time soil visible-near infrared sensor for high resolution soil property mapping. *Geoderma,* **199,** 64-79.

Koestel, J. & Jorda, H. (2014) What determines the strength of preferential transport in undisturbed soil under steady-state flow? *Geoderma,* **217,** 144-160.

Kovacevic, M., Bajat, B. & Gajic, B. (2010) Soil type classification and estimation of soil properties using support vector machines. *Geoderma,* **154,** 340-347.

Krueger, J., Bottcher, J., Schmunk, C. & Bachmann, J. (2016) Soil water repellency and chemical soil properties in a beech forest soil Spatial variability and interrelations. *Geoderma,* **271,** 50-62.

Kuang, B. & Mouazen, A.M. (2011) Calibration of visible and near infrared spectroscopy for soil analysis at the field scale on three European farms. *European Journal of Soil Science,* **62,** 629-636.

Kuang, B. & Mouazen, A.M. (2012) Influence of the number of samples on prediction error of visible and near infrared spectroscopy of selected soil properties at the farm scale. *European Journal of Soil Science,* **63,** 421-429.

Kuang, B.Y. & Mouazen, A.M. (2013) Effect of spiking strategy and ratio on calibration of on-line visible and near infrared soil sensor for measurement in European farms. *Soil & Tillage Research,* **128,** 125-136.

Kuang, X. & Jiao, J.J. (2014) A new equation for the soil water retention curve. *European Journal of Soil Science,* **65,** 584-593.

Kuncoro, P.H., Koga, K., Satta, N. & Muto, Y. (2014) A study on the effect of compaction on transport properties of soil gas and water. II: Soil pore structure indices. *Soil & Tillage Research,* **143,** 180-187.

Lacarce, E., Saby, N.P.A., Martin, M.P., Marchant, B.P., Boulonne, L., Meersmans, J., Jolivet, C., Bispo, A. & Arrouays, D. (2012) Mapping soil Pb stocks and availability in mainland France combining regression trees with robust geostatistics. *Geoderma,* **170,** 359-368.

Lacoste, M., Minasny, B., McBratney, A., Michot, D., Viaud, V. & Walter, C. (2014) High resolution 3D mapping of soil organic carbon in a heterogeneous agricultural landscape. *Geoderma,* **213,** 296-311.

Lafond, J.A., Han, L., Allaire, S.E. & Dutilleul, P. (2012) Multifractal properties of porosity as calculated from computed tomography (CT) images of a sandy soil, in relation to soil gas diffusion and linked soil physical properties. *European Journal of Soil Science,* **63,** 861-873.

Lagacherie, P., Bailly, J.S., Monestiez, P. & Gomez, C. (2012) Using scattered hyperspectral imagery data to map the soil properties of a region. *European Journal of Soil Science,* **63,** 110-119.

Lagacherie, P., Sneep, A.R., Gomez, C., Bacha, S., Coulouma, G., Hamrouni, M.H. & Mekki, I. (2013) Combining Vis-NIR hyperspectral imagery and legacy measured soil profiles to map subsurface soil properties in a Mediterranean area (Cap-Bon, Tunisia). *Geoderma,* **209,** 168-176.

Lai, J.B. & Ren, L. (2016) Estimation of effective hydraulic parameters in heterogeneous soils at field scale. *Geoderma,* **264,** 28-41.

Lang, V., Fuchs, M., Szegi, T., Csorba, A. & Micheli, E. (2016) Deriving World Reference Base Reference Soil Groups from the prospective Global Soil Map product - A case study on major soil types of Africa. *Geoderma,* **263,** 226-233.

Lark, R.M. (2010) Two contrasting spatial processes with a common variogram: inference about spatial models from higher-order statistics. *European Journal of Soil Science,* **61,** 479-492.

Lark, R.M. (2011) Pedometrics. *European Journal of Soil Science,* **62,** 335-336.

Lark, R.M. (2015) Block correlation and the spatial resolution of soil property maps made by kriging. *Geoderma,* **259,** 233-242.

Lark, R.M. & Lapworth, D.J. (2012) Quality measures for soil surveys by lognormal kriging. *Geoderma,* **173,** 231-240.

Lark, R.M. & Lapworth, D.J. (2013) The offset correlation, a novel quality measure for planning geochemical surveys of the soil by kriging. *Geoderma,* **197,** 27-35.

Lark, R.M., Meerschman, E. & Van Meirvenne, M. (2014) A stochastic-geometric model of the variability of soil formed in Pleistocene patterned ground. *Geoderma,* **213,** 533-543.

Lemercier, B., Lacoste, M., Loum, M. & Walter, C. (2012) Extrapolation at regional scale of local soil knowledge using boosted classification trees: A two-step approach. *Geoderma,* **171,** 75-84.

Leue, M., Gerke, H.H. & Ellerbrock, R.H. (2013) Millimetre-scale distribution of organic matter composition at intact biopore and crack surfaces. *European Journal of Soil Science,* **64,** 757-769.

Levi, M.R. & Rasmussen, C. (2014) Covariate selection with iterative principal component analysis for predicting physical soil properties. *Geoderma,* **219,** 46-57.

Li, H.Y., Marchant, B.P. & Webster, R. (2016) Modelling the electrical conductivity of soil in the Yangtze delta in three dimensions. *Geoderma,* **269,** 119-125.

Li, J.W., Richter, D.D., Mendoza, A. & Heine, P. (2010a) Effects of land-use history on soil spatial heterogeneity of macro- and trace elements in the Southern Piedmont USA. *Geoderma,* **156,** 60-73.

Li, P.H., Wang, Q., Endo, T., Zhao, X. & Kakubari, Y. (2010b) Soil organic carbon stock is closely related to aboveground vegetation properties in cold-temperate mountainous forests. *Geoderma,* **154,** 407-415.

Li, Y. (2010) Can the spatial prediction of soil organic matter contents at various sampling scales be improved by using regression kriging with auxiliary information? *Geoderma,* **159,** 63-75.

Li, Y., Fu, X.Q., Liu, X.L., Shen, J.L., Luo, Q., Xiao, R.L., Li, Y.Y., Tong, C.L. & Wu, J.S. (2013) Spatial variability and distribution of N2O emissions from a tea field during the dry season in subtropical central China. *Geoderma,* **193,** 1-12.

Libohova, Z., Wills, S., Odgers, N.P., Ferguson, R., Nesser, R., Thompson, J.A., West, L.T. & Hempel, J.W. (2014) Converting pH 1:1 H2O and 1:2(CaCl2), to 1:5 H2O to contribute to a harmonized global soil database. *Geoderma,* **213,** 544-550.

Liess, M., Glaser, B. & Huwe, B. (2012) Uncertainty in the spatial prediction of soil texture Comparison of regression tree and Random Forest models. *Geoderma,* **170,** 70-79.

Lilburne, L.R., Hewitt, A.E. & Webb, T.W. (2012) Soil and informatics science combine to develop S-map: A new generation soil information system for New Zealand. *Geoderma,* **170,** 232-238.

Lipiec, J., Brzezinska, M., Turski, M., Szarlip, P. & Frac, M. (2015) Wettability and biogeochemical properties of the drilosphere and casts of endogeic earthworms in pear orchard. *Soil & Tillage Research,* **145,** 55-61.

Liu, F., Geng, X.Y., Zhu, A.X., Fraser, W. & Waddell, A. (2012) Soil texture mapping over low relief areas using land surface feedback dynamic patterns extracted from MODIS. *Geoderma,* **171,** 44-52.

Liu, Z.J., Zhou, W., Shen, J.B., He, P., Lei, Q.L. & Liang, G.Q. (2014) A simple assessment on spatial variability of rice yield and selected soil chemical properties of paddy fields in South China. *Geoderma,* **235,** 39-47.

Liu, Z.P., Shao, M.A. & Wang, Y.Q. (2013) Spatial patterns of soil total nitrogen and soil total phosphorus across the entire Loess Plateau region of China. *Geoderma,* **197,** 67-78.

Lopez, R., Gondar, D., Antelo, J., Fiol, S. & Arce, F. (2011) Proton binding on untreated peat and acid-washed peat. *Geoderma,* **164,** 249-253.

Luce, M.S., Whalen, J.K., Ziadi, N. & Zebarth, B.J. (2016) Net nitrogen mineralization enhanced with the addition of nitrogen-rich particulate organic matter. *Geoderma,* **262,** 112-118.

Ludwig, B., Bergstermann, A., Priesack, E. & Flessa, H. (2011) Modelling of crop yields and N2O emissions from silty arable soils with differing tillage in two long-term experiments. *Soil & Tillage Research,* **112,** 114-121.

Ludwig, B., Linsler, D., Hoper, H., Schmidt, H., Piepho, H.P. & Vohland, M. (2016) Pitfalls in the use of middle-infrared spectroscopy: representativeness and ranking criteria for the estimation of soil properties. *Geoderma,* **268,** 165-175.

Mahmood, H.S., Bartholomeus, H.M., Hoogmoed, W.B. & van Henten, E.J. (2013) Evaluation and implementation of vis-NIR spectroscopy models to determine workability. *Soil & Tillage Research,* **134,** 172-179.

Mahmoodabadi, M. & Cerda, A. (2013) WEPP calibration for improved predictions of interrill erosion in semi-arid to arid environments. *Geoderma,* **204,** 75-83.

Malone, B.P., Minasny, B., Odgers, N.P. & McBratney, A.B. (2014) Using model averaging to combine soil property rasters from legacy soil maps and from point data. *Geoderma,* **232,** 34-44.

Mansuy, N., Thiffault, E., Pare, D., Bernier, P., Guindon, L., Villemaire, P., Poirier, V. & Beaudoin, A. (2014) Digital mapping of soil properties in Canadian managed forests at 250 m of resolution using the k-nearest neighbor method. *Geoderma,* **235,** 59-73.

Marchant, B.P., Saby, N.P.A., Jolivet, C.C., Arrouays, D. & Lark, R.M. (2011) Spatial prediction of soil properties with copulas. *Geoderma,* **162,** 327-334.

Mareschal, L., Nzila, J.D.D., Turpault, M.P., M'Bou, A.T., Mazoumbou, J.C., Bouillet, J.P., Ranger, J. & Laclau, J.P. (2011) Mineralogical and physico-chemical properties of Ferralic Arenosols derived from unconsolidated Plio-Pleistocenic deposits in the coastal plains of Congo. *Geoderma,* **162,** 159-170.

Marin-Castro, B.E., Geissert, D., Negrete-Yankelevich, S. & Chavez, A.G.T. (2016) Spatial distribution of hydraulic conductivity in soils of secondary tropical montane cloud forests and shade coffee agroecosystems. *Geoderma,* **283,** 57-67.

Marin-Gonzalez, O., Kuang, B.Y., Quraishi, M.Z., Munoz-Garcia, M.A. & Mouazen, A.M. (2013) On-line measurement of soil properties without direct spectral response in near infrared spectral range. *Soil & Tillage Research,* **132,** 21-29.

Marques, J., Siqueira, D.S., Camargo, L.A., Teixeira, D.D.B., Barron, V. & Torrent, J. (2014) Magnetic susceptibility and diffuse reflectance spectroscopy to characterize the spatial variability of soil properties in a Brazilian Haplustalf. *Geoderma,* **219,** 63-71.

Martin, M., Bonifacio, E., Hossain, K.M.J., Huq, S.M.I. & Barberis, E. (2014) Arsenic fixation and mobilization in the soils of the Ganges and Meghna floodplains. Impact of pedoenvironmental properties. *Geoderma,* **228,** 132-141.

Mashimbye, Z.E., de Clercq, W.P. & Van Niekerk, A. (2014) An evaluation of digital elevation models (DEMs) for delineating land components. *Geoderma,* **213,** 312-319.

Maynard, J.J. & Johnson, M.G. (2014) Scale-dependency of LiDAR derived terrain attributes in quantitative soil-landscape modeling: Effects of grid resolution vs. neighborhood extent. *Geoderma,* **230,** 29-40.

Mikhailova, E.A., Altememe, A.H., Bawazir, A.A., Chandler, R.D., Cope, M.P., Post, C.J., Stiglitz, R.Y., Zurqani, H.A. & Schlautman, M.A. (2016) Comparing soil carbon estimates in glaciated soils at a farm scale using geospatial analysis of field and SSURGO data. *Geoderma,* **281,** 119-126.

Millan, H., Tarquis, A.M., Perez, L.D., Mato, J. & Gonzalez-Posada, M. (2012) Spatial variability patterns of some Vertisol properties at a field scale using standardized data. *Soil & Tillage Research,* **120,** 76-84.

Miller, B.A., Koszinski, S., Hierold, W., Rogasik, H., Schroder, B., Van Oost, K., Wehrhan, M. & Sommer, M. (2016) Towards mapping soil carbon landscapes: Issues of sampling scale and transferability. *Soil & Tillage Research,* **156,** 194-208.

Miller, B.A., Koszinski, S., Wehrhan, M. & Sommer, M. (2015) Impact of multi-scale predictor selection for modeling soil properties. *Geoderma,* **239,** 97-106.

Miller, B.A. & Schaetzl, R.J. (2014) The historical role of base maps in soil geography. *Geoderma,* **230,** 329-339.

Miller, B.A. & Schaetzl, R.J. (2016) History of soil geography in the context of scale. *Geoderma,* **264,** 284-300.

Minasny, B., McBratney, A.B., Brough, D.M. & Jacquier, D. (2011) Models relating soil pH measurements in water and calcium chloride that incorporate electrolyte concentration. *European Journal of Soil Science,* **62,** 728-732.

Minasny, B., Vrugt, J.A. & McBratney, A.B. (2011) Confronting uncertainty in model-based geostatistics using Markov Chain Monte Carlo simulation. *Geoderma,* **163,** 150-162.

Mora, J.L. & Lazaro, R. (2014) Seasonal changes in bulk density under semiarid patchy vegetation: the soil beats. *Geoderma,* **235,** 30-38.

Moral, F.J., Terron, J.M. & da Silva, J.R.M. (2010) Delineation of management zones using mobile measurements of soil apparent electrical conductivity and multivariate geostatistical techniques. *Soil & Tillage Research,* **106,** 335-343.

Mordhorst, A., Peth, S. & Horn, R. (2014) Influence of mechanical loading on static and dynamic CO2 efflux on differently textured and managed Luvisols. *Geoderma,* **219,** 1-13.

Mouazen, A.M., Alhwaimel, S.A., Kuang, B. & Waine, T. (2014) Multiple on-line soil sensors and data fusion approach for delineation of water holding capacity zones for site specific irrigation. *Soil & Tillage Research,* **143,** 95-105.

Mouazen, A.M. & Kuang, B. (2016) On-line visible and near infrared spectroscopy for in-field phosphorous management. *Soil & Tillage Research,* **155,** 471-477.

Mouazen, A.M., Kuang, B., De Baerdemaeker, J. & Ramon, H. (2010) Comparison among principal component, partial least squares and back propagation neural network analyses for accuracy of measurement of selected soil properties with visible and near infrared spectroscopy. *Geoderma,* **158,** 23-31.

Mulder, V.L., de Bruin, S., Schaepman, M.E. & Mayr, T.R. (2011) The use of remote sensing in soil and terrain mapping - A review. *Geoderma,* **162,** 1-19.

Mulder, V.L., Lacoste, M., Richer-de-Forges, A.C., Martin, M.P. & Arrouays, D. (2016) National versus global modelling the 3D distribution of soil organic carbon in mainland France. *Geoderma,* **263,** 16-34.

Myers, D.B., Kitchen, N.R., Sudduth, K.A., Miles, R.J., Sadler, E.J. & Grunwald, S. (2011) Peak functions for modeling high resolution soil profile data. *Geoderma,* **166,** 74-83.

Naderi-Boldaji, M., Alimardani, R., Hemmat, A., Sharifi, A., Keyhani, A., Tekeste, M.Z. & Keller, T. (2014) 3D finite element simulation of a single-tip horizontal penetrometer-soil interaction. Part II: Soil bin verification of the model in a clay-loam soil. *Soil & Tillage Research,* **144,** 211-219.

Naderi-Boldaji, M., Sharifi, A., Alimardani, R., Hemmat, A., Keyhani, A., Loonstra, E.H., Weisskopf, P., Stettler, M. & Keller, T. (2013) Use of a triple-sensor fusion system for on-the-go measurement of soil compaction. *Soil & Tillage Research,* **128,** 44-53.

Nano, C.C.U., Nicolardot, B., Quinche, M., Munier-Jolain, N. & Ubertosi, M. (2016) Effects of integrated weed management based cropping systems on the water retention of a silty clay loam soil. *Soil & Tillage Research,* **156,** 74-82.

Nauman, T.W. & Thompson, J.A. (2014) Semi-automated disaggregation of conventional soil maps using knowledge driven data mining and classification trees. *Geoderma,* **213,** 385-399.

Nauman, T.W., Thompson, J.A., Teets, S.J., Dilliplane, T.A., Bell, J.W., Connolly, S.J., Liebermann, H.J. & Yoast, K.M. (2015) Ghosts of the forest: Mapping pedomemory to guide forest restoration. *Geoderma,* **247,** 51-64.

Nawar, S., Buddenbaum, H., Hill, J., Kozak, J. & Mouazen, A.M. (2016) Estimating the soil clay content and organic matter by means of different calibration methods of vis-NIR diffuse reflectance spectroscopy. *Soil & Tillage Research,* **155,** 510-522.

Neely, H.L., Morgan, C.L.S., Hallmark, C.T., McInnes, K.J. & Molling, C.C. (2016) Apparent electrical conductivity response to spatially variable vertisol properties. *Geoderma,* **263,** 168-175.

Neris, J., Doerr, S.H., Tejedor, M., Jimenez, C. & Hernandez-Moreno, J.M. (2014) Thermal analysis as a predictor for hydrological parameters of fire-affected soils. *Geoderma,* **235,** 240-249.

Neyshabouri, M.R., Kazemi, Z., Oustan, S. & Moghaddam, M. (2014) PTFs for predicting LLWR from various soil attributes including cementing agents. *Geoderma,* **226,** 179-187.

Nezhad, M.T.K., Mohammadi, K., Gholami, A., Hani, A. & Shariati, M.S. (2014) Cadmium and mercury in topsoils of Babagorogor watershed, western Iran: Distribution, relationship with soil characteristics and multivariate analysis of contamination sources. *Geoderma,* **219,** 177-185.

O'Brien, S.L., Jastrow, J.D., Grimley, D.A. & Gonzalez-Meler, M.A. (2015) Edaphic controls on soil organic carbon stocks in restored grasslands. *Geoderma,* **251,** 117-123.

O'Rourke, S.M., Stockmann, U., Holden, N.M., McBratney, A.B. & Minasny, B. (2016) An assessment of model averaging to improve predictive power of portable vis-NIR and XRF for the determination of agronomic soil properties. *Geoderma,* **279,** 31-44.

Odgers, N.P., McBratney, A.B. & Minasny, B. (2011a) Bottom-up digital soil mapping. I. Soil layer classes. *Geoderma,* **163,** 38-44.

Odgers, N.P., McBratney, A.B. & Minasny, B. (2011b) Bottom-up digital soil mapping. II. Soil series classes. *Geoderma,* **163,** 30-37.

Odgers, N.P., McBratney, A.B. & Minasny, B. (2015) Digital soil property mapping and uncertainty estimation using soil class probability rasters. *Geoderma,* **237,** 190-198.

Ogg, C.M., Wilson, M.A., Reed, J.M. & Gulley, C.D. (2013) Sediment provenance and geomorphic development of soils in a blackwater ecosystem, South Carolina Coastal Plain. *Geoderma,* **192,** 394-406.

Ohno, T. & Amirbahman, A. (2010) Phosphorus availability in boreal forest soils: A geochemical and nutrient uptake modeling approach. *Geoderma,* **155,** 46-54.

Okada, E., Costa, J.L., Bedmar, F., Barbagelata, P., Irizar, A. & Rampoldi, E.A. (2014) Effect of conventional and no-till practices on solute transport in long term field trials. *Soil & Tillage Research,* **142,** 8-14.

Ortiz, B.V., Perry, C., Goovaerts, P., Vellidis, G. & Sullivan, D. (2010) Geostatistical modeling of the spatial variability and risk areas of southern root-knot nematodes in relation to soil properties. *Geoderma,* **156,** 243-252.

Orton, T.G., Pringle, M.J., Allen, D.E., Dalal, R.C. & Bishop, T.F.A. (2015) A geostatistical method to account for the number of aliquots in composite samples for normal and lognormal random variables. *European Journal of Soil Science,* **66,** 1023-1032.

Orton, T.G., Pringle, M.J. & Bishop, T.F.A. (2016) A one-step approach for modelling and mapping soil properties based on profile data sampled over varying depth intervals. *Geoderma,* **262,** 174-186.

Otto, R., Silva, A.P., Franco, H.C.J., Oliveira, E.C.A. & Trivelin, P.C.O. (2011) High soil penetration resistance reduces sugarcane root system development. *Soil & Tillage Research,* **117,** 201-210.

Ouerghemmi, W., Gomez, C., Naceur, S. & Lagacherie, P. (2011) Applying blind source separation on hyperspectral data for clay content estimation over partially vegetated surfaces. *Geoderma,* **163,** 227-237.

Pan, Y.Y., Bonten, L.T.C., Koopmans, G.F., Song, J., Luo, Y.M., Temminghoff, E.J.M. & Comans, R.N.J. (2016) Solubility of trace metals in two contaminated paddy soils exposed to alternating flooding and drainage. *Geoderma,* **261,** 59-69.

Papanicolaou, A.N., Elhakeem, M., Wilson, C.G., Burras, C.L., West, L.T., Lin, H., Clark, B. & Oneal, B.E. (2015) Spatial variability of saturated hydraulic conductivity at the hillslope scale: Understanding the role of land management and erosional effect. *Geoderma,* **243,** 58-68.

Paradelo, M., Norgaard, T., Moldrup, P., Ferre, T.P.A., Kumari, K., Arthur, E. & de Jonge, L.W. (2015) Prediction of the glyphosate sorption coefficient across two loamy agricultural fields. *Geoderma,* **259,** 224-232.

Pare, N., Andrieux, P., Louchart, X., Biarnes, A. & Voltz, M. (2011) Predicting the spatio-temporal dynamic of soil surface characteristics after tillage. *Soil & Tillage Research,* **114,** 135-145.

Parent, L.E., de Almeida, C.X., Hernandes, A., Egozcue, J.J., Gulser, C., Bolinder, M.A., Katterer, T., Andren, O., Parent, S.E., Anctil, F., Centurion, J.F. & Natale, W. (2012) Compositional analysis for an unbiased measure of soil aggregation. *Geoderma,* **179,** 123-131.

Parker, S.S., Seabloom, E.W. & Schimel, J.P. (2012) Grassland community composition drives small-scale spatial patterns in soil properties and processes. *Geoderma,* **170,** 269-279.

Pascucci, S., Casa, R., Belviso, C., Palombo, A., Pignatti, S. & Castaldi, F. (2014) Estimation of soil organic carbon from airborne hyperspectral thermal infrared data: a case study. *European Journal of Soil Science,* **65,** 865-875.

Paul, R. & Cressie, N. (2011) Lognormal block kriging for contaminated soil. *European Journal of Soil Science,* **62,** 337-345.

Paz-Ferreiro, J., Vazquez, E.V. & Miranda, J.G.V. (2010) Assessing soil particle-size distribution on experimental plots with similar texture under different management systems using multifractal parameters. *Geoderma,* **160,** 47-56.

Penne, C., Ahrends, B., Deurer, M. & Bottcher, J. (2010) The impact of the canopy structure on the spatial variability in forest floor carbon stocks. *Geoderma,* **158,** 282-297.

Perez, L.D., Millan, H. & Gonzalez-Posada, M. (2010) Spatial complexity of soil plow layer penetrometer resistance as influenced by sugarcane harvesting: A prefractal approach. *Soil & Tillage Research,* **110,** 77-86.

Peth, S., Rostek, J., Zink, A., Mordhorst, A. & Horn, R. (2010) Soil testing of dynamic deformation processes of arable soils. *Soil & Tillage Research,* **106,** 317-328.

Pinuela, J., Alvarez, A., Andina, D., Heck, R.J. & Tarquis, A.M. (2010) Quantifying a soil pore distribution from 3D images: Multifractal spectrum through wavelet approach. *Geoderma,* **155,** 203-210.

Piotrowska, A. & Dlugosz, J. (2012) Spatio-temporal variability of microbial biomass content and activities related to some physicochemical properties of Luvisols. *Geoderma,* **173,** 199-208.

Poggio, L. & Gimona, A. (2014) National scale 3D modelling of soil organic carbon stocks with uncertainty propagation - An example from Scotland. *Geoderma,* **232,** 284-299.

Poggio, L., Gimona, A. & Brewer, M.J. (2013) Regional scale mapping of soil properties and their uncertainty with a large number of satellite-derived covariates. *Geoderma,* **209,** 1-14.

Poggio, L., Gimona, A., Brown, I. & Castellazzi, M. (2010) Soil available water capacity interpolation and spatial uncertainty modelling at multiple geographical extents. *Geoderma,* **160,** 175-188.

Poggio, L., Gimona, A., Spezia, L. & Brewer, M.J. (2016) Bayesian spatial modelling of soil properties and their uncertainty: The example of soil organic matter in Scotland using R-INLA. *Geoderma,* **277,** 69-82.

Pulido Moncada, M., Gabriels, D. & Cornelis, W.M. (2014) Data-driven analysis of soil quality indicators using limited data. *Geoderma,* **235,** 271-278.

Quraishi, M.Z. & Mouazen, A.M. (2013) A prototype sensor for the assessment of soil bulk density. *Soil & Tillage Research,* **134,** 97-110.

Ramirez-Guinart, O., Vidal, M. & Rigol, A. (2016) Univariate and multivariate analysis to elucidate the soil properties governing americium sorption in soils. *Geoderma,* **269,** 19-26.

Ramos, T.B., Horta, A., Goncalves, M.C., Martins, J.C. & Pereira, L.S. (2014) Development of ternary diagrams for estimating water retention properties using geostatistical approaches. *Geoderma,* **230,** 229-242.

Rawlins, B.G., Turner, G., Wragg, J., McLachlan, P. & Lark, R.M. (2015) An improved method for measurement of soil aggregate stability using laser granulometry applied at regional scale. *European Journal of Soil Science,* **66,** 604-614.

Rejman, J., Iglik, I., Paluszek, J. & Rodzik, J. (2014) Soil redistribution and crop productivity in loess areas (Lublin Upland, Poland). *Soil & Tillage Research,* **143,** 77-84.

Ren, Z.L., Sivry, Y., Dai, J., Tharaud, M., Cordier, L. & Benedetti, M.F. (2015) Multi-element stable isotopic dilution and multi-surface modelling to assess the speciation and reactivity of cadmium and copper in soil. *European Journal of Soil Science,* **66,** 973-982.

Reyhanitabar, A. & Gilkes, R.J. (2010) Kinetics of DTPA extraction of zinc from calcareous soils. *Geoderma,* **154,** 289-293.

Rieckh, H., Gerke, H.H. & Sommer, M. (2012) Hydraulic properties of characteristic horizons depending on relief position and structure in a hummocky glacial soil landscape. *Soil & Tillage Research,* **125,** 123-131.

Rienzner, M. & Gandolfi, C. (2014) Investigation of spatial and temporal variability of saturated soil hydraulic conductivity at the field-scale. *Soil & Tillage Research,* **135,** 28-40.

Rodrigues, F.A., Bramley, R.G.V. & Gobbett, D.L. (2015) Proximal soil sensing for Precision Agriculture: Simultaneous use of electromagnetic induction and gamma radiometrics in contrasting soils. *Geoderma,* **243,** 183-195.

Roger, A., Libohova, Z., Rossier, N., Joost, S., Maltas, A., Frossard, E. & Sinaj, S. (2014) Spatial variability of soil phosphorus in the Fribourg canton, Switzerland. *Geoderma,* **217,** 26-36.

Romano, N. & Nasta, P. (2016) How effective is bimodal soil hydraulic characterization? Functional evaluations for predictions of soil water balance. *European Journal of Soil Science,* **67,** 523-535.

Ros, G.H., Temminghoff, E.J.M. & Hoffland, E. (2011) Nitrogen mineralization: a review and meta-analysis of the predictive value of soil tests. *European Journal of Soil Science,* **62,** 162-173.

Rossel, R.A.V. & Behrens, T. (2010) Using data mining to model and interpret soil diffuse reflectance spectra. *Geoderma,* **158,** 46-54.

Rossel, R.A.V., Chappell, A., de Caritat, P. & McKenzie, N.J. (2011) On the soil information content of visible-near infrared reflectance spectra. *European Journal of Soil Science,* **62,** 442-453.

Rossel, R.A.V. & Webster, R. (2012) Predicting soil properties from the Australian soil visible-near infrared spectroscopic database. *European Journal of Soil Science,* **63,** 848-860.

Rossiter, D.G., Liu, J., Carlisle, S. & Zhu, A.X. (2015) Can citizen science assist digital soil mapping? *Geoderma,* **259,** 71-80.

Ruckamp, D., Martius, C., Bornemann, L., Kurzatkowski, D., Naval, L.P. & Amelung, W. (2012) Soil genesis and heterogeneity of phosphorus forms and carbon below mounds inhabited by primary and secondary termites. *Geoderma,* **170,** 239-250.

Ryken, N., Al-Barri, B., Taylor, A., Blake, W., Maenhout, P., Sleutel, S., Tack, F.M.G., Dierick, M., Bode, S., Boeckx, P. & Verdoodt, A. (2016) Quantifying the spatial variation of Be-7 depth distributions towards improved erosion rate estimations. *Geoderma,* **269,** 10-18.

Saby, N.P.A., Marchant, B.P., Lark, R.M., Jolivet, C.C. & Arrouays, D. (2011) Robust geostatistical prediction of trace elements across France. *Geoderma,* **162,** 303-311.

Salley, S.W., Sleezer, R.O., Bergstrom, R.M., Martin, P.H. & Kelly, E.F. (2016) A long-term analysis of the historical dry boundary for the Great Plains of North America: Implications of climatic variability and climatic change on temporal and spatial patterns in soil moisture. *Geoderma,* **274,** 104-113.

Samuel-Rosa, A., Heuvelink, G.B.M., Vasques, G.M. & Anjos, L.H.C. (2015) Do more detailed environmental covariates deliver more accurate soil maps? *Geoderma,* **243,** 214-227.

Samyn, K., Cerdan, O., Grandjean, G., Cochery, R., Bernardie, S. & Bitri, A. (2012) Assessment of vulnerability to erosion: Digital mapping of a loess cover thickness and stiffness using spectral analysis of seismic surface-waves. *Geoderma,* **173,** 162-172.

Sarkhot, D.V., Grunwald, S., Ge, Y. & Morgan, C.L.S. (2011) Comparison and detection of total and available soil carbon fractions using visible/near infrared diffuse reflectance spectroscopy. *Geoderma,* **164,** 22-32.

Savvides, A., Corstanje, R., Baxter, S.J., Rawlins, B.G. & Lark, R.M. (2010) The relationship between diffuse spectral reflectance of the soil and its cation exchange capacity is scale-dependent. *Geoderma,* **154,** 353-358.

Schjonning, P. & Thomsen, I.K. (2013) Shallow tillage effects on soil properties for temperate-region hard-setting soils. *Soil & Tillage Research,* **132,** 12-20.

Schmidt, K., Behrens, T., Daumann, J., Ramirez-Lopez, L., Werban, U., Dietrich, P. & Scholten, T. (2014) A comparison of calibration sampling schemes at the field scale. *Geoderma,* **232,** 243-256.

Seger, M., Guerin, R., Frison, A., Bourennane, H., Richard, G. & Cousin, I. (2014) A 3D electrical resistivity tomography survey to characterise the structure of a albeluvic tonguing horizon composed of distinct elementary pedological volumes. *Geoderma,* **219,** 168-176.

Selim, H.M., Newman, A., Zhang, L.Y., Arceneaux, A., Tubana, B. & Gaston, L.A. (2016) Distributions of organic carbon and related parameters in a Louisiana sugarcane soil. *Soil & Tillage Research,* **155,** 401-411.

Sequeira, C.H., Wills, S.A., Seybold, C.A. & West, L.T. (2014) Predicting soil bulk density for incomplete databases. *Geoderma,* **213,** 64-73.

Shi, W.J., Liu, J.Y., Du, Z.P., Stein, A. & Yue, T.X. (2011) Surface modelling of soil properties based on land use information. *Geoderma,* **162,** 347-357.

Shi, X.H., Yang, X.M., Drury, C.F., Reynolds, W.D., McLaughlin, N.B. & Zhang, X.P. (2012) \Impact of ridge tillage on soil organic carbon and selected physical properties of a clay loam in southwestern Ontario. *Soil & Tillage Research,* **120,** 1-7.

Shi, Z., Ji, W., Rossel, R.A.V., Chen, S. & Zhou, Y. (2015) Prediction of soil organic matter using a spatially constrained local partial least squares regression and the Chinese vis-NIR spectral library. *European Journal of Soil Science,* **66,** 679-687.

Shin, H.C., Taherzadeh, S., Attenborough, K., Whalley, W.R. & Watts, C.W. (2013) Non-invasive characterization of pore-related and elastic properties of soils in linear Biot-Stoll theory using acoustic-to-seismic coupling. *European Journal of Soil Science,* **64,** 308-323.

Shirani, H., Habibi, M., Besalatpour, A.A. & Esfandiarpour, I. (2015) Determining the features influencing physical quality of calcareous soils in a semiarid region of Iran using a hybrid PSO-DT algorithm. *Geoderma,* **259,** 1-11.

Shukla, A.K., Behera, S.K., Lenka, N.K., Tiwari, P.K., Prakash, C., Malik, R.S., Sinha, N.K., Singh, V.K., Patra, A.K. & Chaudhary, S.K. (2016) Spatial variability of soil micronutrients in the intensively cultivated Trans-Gangetic Plains of India. *Soil & Tillage Research,* **163,** 282-289.

Singh, B., Farenhorst, A., Gaultier, J., Pennock, D., Degenhardt, D. & McQueen, R. (2014) Soil characteristics and herbicide sorption coefficients in 140 soil profiles of two irregular undulating to hummocky terrains of western Canada. *Geoderma,* **232,** 107-116.

Singh, H.V. & Thompson, A.M. (2016) Effect of antecedent soil moisture content on soil critical shear stress in agricultural watersheds. *Geoderma,* **262,** 165-173.

Siqueira, D.S., Marques, J. & Pereira, G.T. (2010) The use of landforms to predict the variability of soil and orange attributes. *Geoderma,* **155,** 55-66.

Soderstrom, M. & Eriksson, J. (2013) Gamma-ray spectrometry and geological maps as tools for cadmium risk assessment in arable soils. *Geoderma,* **192,** 323-334.

Soriano-Disla, J.M., Gomez, I., Guerrero, C., Navarro-Pedreno, J. & Garcia-Orenes, F. (2010) The potential of NIR spectroscopy to predict stability parameters in sewage sludge and derived compost. *Geoderma,* **158,** 93-100.

Spielvogel, S., Prietzel, J. & Kogel-Knabner, I. (2016) Stand scale variability of topsoil organic matter composition in a high-elevation Norway spruce forest ecosystem. *Geoderma,* **267,** 112-122.

Steffens, M. & Buddenbaum, H. (2013) Laboratory imaging spectroscopy of a stagnic Luvisol profile - High resolution soil characterisation, classification and mapping of elemental concentrations. *Geoderma,* **195,** 122-132.

Stevens, A., Udelhoven, T., Denis, A., Tychon, B., Lioy, R., Hoffmann, L. & van Wesemael, B. (2010) Measuring soil organic carbon in croplands at regional scale using airborne imaging spectroscopy. *Geoderma,* **158,** 32-45.

Stevens, F., Bogaert, P. & van Wesemael, B. (2015a) Detecting and quantifying field-related spatial variation of soil organic carbon using mixed-effect models and airborne imagery. *Geoderma,* **259,** 93-103.

Stevens, F., Bogaert, P. & van Wesemael, B. (2015b) Spatial filtering of a legacy dataset to characterize relationships between soil organic carbon and soil texture. *Geoderma,* **237,** 224-236.

Stevenson, B.A., McNeill, S. & Hewitt, A.E. (2015) Characterising soil quality clusters in relation to land use and soil order in New Zealand: An application of the phenoform concept. *Geoderma,* **239,** 135-142.

Stockmann, U., Malone, B.P., McBratney, A.B. & Minasny, B. (2015) Landscape-scale exploratory radiometric mapping using proximal soil sensing. *Geoderma,* **239,** 115-129.

Sulaeman, Y., Minasny, B., McBratney, A.B., Sarwani, M. & Sutandi, A. (2013) Harmonizing legacy soil data for digital soil mapping in Indonesia. *Geoderma,* **192,** 77-85.

Sun, W., Minasny, B. & McBratney, A. (2012) Analysis and prediction of soil properties using local regression-kriging. *Geoderma,* **171,** 16-23.

Sun, X.L., Wu, S.C., Wang, H.L., Zhao, Y.G., Zhang, G.L., Man, Y.B. & Wong, M.H. (2013) Dealing with spatial outliers and mapping uncertainty for evaluating the effects of urbanization on soil: A case study of soil pH and particle fractions in Hong Kong. *Geoderma,* **195,** 220-233.

Sun, X.L., Wu, Y.J., Lou, Y.L., Wang, H.L., Zhang, C., Zhao, Y.G. & Zhang, G.L. (2015) Updating digital soil maps with new data: a case study of soil organic matter in Jiangsu, China. *European Journal of Soil Science,* **66,** 1012-1022.

Suuster, E., Ritz, C., Roostalu, H., Kolli, R. & Astover, A. (2012) Modelling soil organic carbon concentration of mineral soils in arable land using legacy soil data. *European Journal of Soil Science,* **63,** 351-359.

Suuster, E., Ritz, C., Roostalu, H., Reintam, E., Kolli, R. & Astover, A. (2011) Soil bulk density pedotransfer functions of the humus horizon in arable soils. *Geoderma,* **163,** 74-82.

Szopka, K., Karczewska, A., Jezierski, P. & Kabala, C. (2013) Spatial distribution of lead in the surface layers of mountain forest soils, an example from the Karkonosze National Park, Poland. *Geoderma,* **192,** 259-268.

Taalab, K., Corstanje, R., Mayr, T.M., Whelan, M.J. & Creamer, R.E. (2015a) The application of expert knowledge in Bayesian networks to predict soil bulk density at the landscape scale. *European Journal of Soil Science,* **66,** 930-941.

Taalab, K., Corstanje, R., Zawadzka, J., Mayr, T., Whelan, M.J., Hannam, J.A. & Creamer, R. (2015b) On the application of Bayesian Networks in Digital Soil Mapping. *Geoderma,* **259,** 134-148.

Taconet, O., Vannier, E. & Le Hegarat-Mascle, S. (2010) A contour-based approach for clods identification and characterization on a soil surface. *Soil & Tillage Research,* **109,** 123-132.

Taghizadeh-Mehrjardi, R., Minasny, B., Sarmadian, F. & Malone, B.P. (2014) Digital mapping of soil salinity in Ardakan region, central Iran. *Geoderma,* **213,** 15-28.

Taghizadeh-mehrjardi, R., Toomanian, N., Khavaninzadeh, A.R., Jafari, A. & Triantafilis, J. (2016) Predicting and mapping of soil particle-size fractions with adaptive neuro-fuzzy inference and ant colony optimization in central Iran. *European Journal of Soil Science,* **67,** 707-725.

Takawira, A., Gwenzi, W. & Nyamugafata, P. (2014) Does hydrocarbon contamination induce water repellency and changes in hydraulic properties in inherently wettable tropical sandy soils? *Geoderma,* **235,** 279-289.

Tanikawa, T., Yamashita, N., Aizawa, S., Ohnuki, Y., Yoshinaga, S. & Takahashi, M. (2013) Soil sulfur content and its spatial distribution in a small catchment covered by volcanic soil in the montane zone of central Japan. *Geoderma,* **197,** 1-8.

Terra, F.S., Dematte, J.A.M. & Rossel, R.A.V. (2015) Spectral libraries for quantitative analyses of tropical Brazilian soils: Comparing vis-NIR and mid-IR reflectance data. *Geoderma,* **255,** 81-93.

Tesfahunegn, G.B., Tamene, L. & Vlek, P.L.G. (2011) Catchment-scale spatial variability of soil properties and implications on site-specific soil management in northern Ethiopia. *Soil & Tillage Research,* **117,** 124-139.

Thiffault, E., Pare, D., Guindon, L., Beaudoin, A., Brais, S., Leduc, A. & Michel, J.P. (2013) Assessing forest soil base cation status and availability using lake and stream sediment geochemistry: A case study in Quebec (Canada). *Geoderma,* **211,** 39-50.

Thomas, M., Clifford, D., Bartley, R., Philip, S., Brough, D., Gregory, L., Willis, R. & Glover, M. (2015) Putting regional digital soil mapping into practice in Tropical Northern Australia. *Geoderma,* **241,** 145-157.

Torrent, J., Liu, Q.S. & Barron, V. (2010) Magnetic minerals in Calcic Luvisols (Chromic) developed in a warm Mediterranean region of Spain: Origin and paleoenvironmental significance. *Geoderma,* **154,** 465-472.

Triantafilis, J., Gibbs, I. & Earl, N. (2013) Digital soil pattern recognition in the lower Namoi valley using numerical clustering of gamma-ray spectrometry data. *Geoderma,* **192,** 407-421.

Triantafilis, J. & Santos, F.A.M. (2013) Electromagnetic conductivity imaging (EMCI) of soil using a DUALEM-421 and inversion modelling software (EM4Soil). *Geoderma,* **211,** 28-38.

Truong, P.N. & Heuvelink, G.B.M. (2013) Uncertainty quantification of soil property maps with statistical expert elicitation. *Geoderma,* **202,** 142-152.

Vagen, T.G., Winowiecki, L.A., Tondoh, J.E., Desta, L.T. & Gumbricht, T. (2016) Mapping of soil properties and land degradation risk in Africa using MODIS reflectance. *Geoderma,* **263,** 216-225.

Van Meirvenne, M., Islam, M.M., De Smedt, P., Meerschman, E., Van de Vijver, E. & Saey, T. (2013) Key variables for the identification of soil management classes in the aeolian landscapes of north-west Europe. *Geoderma,* **199,** 99-105.

Vasat, R., Kodesova, R., Boruvka, L., Klement, A., Jaksik, O. & Gholizadeh, A. (2014) Consideration of peak parameters derived from continuum-removed spectra to predict extractable nutrients in soils with visible and near-infrared diffuse reflectance spectroscopy (VNIR-DRS). *Geoderma,* **232,** 208-218.

Vaz, C.M.P., Manieri, J.M., de Maria, I.C. & Tuller, M. (2011) Modeling and correction of soil penetration resistance for varying soil water content. *Geoderma,* **166,** 92-101.

Vendrame, P.R.S., Marchao, R.L., Brunet, D. & Becquer, T. (2012) The potential of NIR spectroscopy to predict soil texture and mineralogy in Cerrado Latosols. *European Journal of Soil Science,* **63,** 743-753.

Visconti, F., de Paz, J.M. & Rubio, J.L. (2010) An empirical equation to calculate soil solution electrical conductivity at 25 degrees C from major ion concentrations. *European Journal of Soil Science,* **61,** 980-993.

Vogel, S. & Marker, M. (2011) Characterization of the pre-AD 79 Roman paleosol south of Pompeii (Italy): Correlation between soil parameter values and paleo-topography. *Geoderma,* **160,** 548-558.

Vohland, M., Besold, J., Hill, J. & Frund, H.C. (2011) Comparing different multivariate calibration methods for the determination of soil organic carbon pools with visible to near infrared spectroscopy. *Geoderma,* **166,** 198-205.

Vohland, M. & Emmerling, C. (2011) Determination of total soil organic C and hot water-extractable C from VIS-NIR soil reflectance with partial least squares regression and spectral feature selection techniques. *European Journal of Soil Science,* **62,** 598-606.

Vohland, M., Ludwig, M., Thiele-Bruhn, S. & Ludwig, B. (2014) Determination of soil properties with visible to near- and mid-infrared spectroscopy: Effects of spectral variable selection. *Geoderma,* **223,** 88-96.

Wahren, F.T., Julich, S., Nunes, J.P., Gonzalez-Pelayo, O., Hawtree, D., Feger, K.H. & Keizer, J.J. (2016) Combining digital soil mapping and hydrological modeling in a data scarce watershed in north-central Portugal. *Geoderma,* **264,** 350-362.

Wang, C., Yang, Z.F., Yuan, X.Y., Browne, P., Chen, L.X. & Ji, J.F. (2013) The influences of soil properties on Cu and Zn availability in soil and their transfer to wheat (Triticum aestivum L.) in the Yangtze River delta region, China. *Geoderma,* **193,** 131-139.

Wang, D.D., Chakraborty, S., Weindorf, D.C., Li, B., Sharma, A., Paul, S. & Ali, M.N. (2015) Synthesized use of VisNIR DRS and PXRF for soil characterization: Total carbon and total nitrogen. *Geoderma,* **243,** 157-167.

Wang, T.J., Chen, X.H., Tang, A.M. & Cui, Y.J. (2014) On the use of the similar media concept for scaling soil air permeability. *Geoderma,* **235,** 154-162.

Wang, X.Y., Westbrook, C. & Bedard-Haughn, A. (2016) Effect of mineral horizons on spatial distribution of soil properties and N cycling in a mountain peatland. *Geoderma,* **273,** 73-82.

Wang, Y., Zhang, J.H. & Zhang, Z.H. (2015) Influences of intensive tillage on water-stable aggregate distribution on a steep hillslope. *Soil & Tillage Research,* **151,** 82-92.

Watt, M.S. & Palmer, D.J. (2012) Use of regression kriging to develop a Carbon:Nitrogen ratio surface for New Zealand. *Geoderma,* **183,** 49-57.

Weindorf, D.C., Chakraborty, S., Herrero, J., Li, B., Castaneda, C. & Choudhury, A. (2016) Simultaneous assessment of key properties of arid soil by combined PXRF and Vis-NIR data. *European Journal of Soil Science,* **67,** 173-183.

Wells, T., Hancock, G.R., Dever, C. & Murphy, D. (2012) Prediction of vertical soil organic carbon profiles using soil properties and environmental tracer data at an untilled site. *Geoderma,* **170,** 337-346.

Wetterlind, J., Piikki, K., Stenberg, B. & Soderstrom, M. (2015) Exploring the predictability of soil texture and organic matter content with a commercial integrated soil profiling tool. *European Journal of Soil Science,* **66,** 631-638.

Wetterlind, J. & Stenberg, B. (2010) Near-infrared spectroscopy for within-field soil characterization: small local calibrations compared with national libraries spiked with local samples. *European Journal of Soil Science,* **61,** 823-843.

Wight, J.P., Ashworth, A.J. & Allen, F.L. (2016) Organic substrate, clay type, texture, and water influence on NIR carbon measurements. *Geoderma,* **261,** 36-43.

Wijewardane, N.K., Ge, Y.F. & Morgan, C.L.S. (2016) Moisture insensitive prediction of soil properties from VNIR reflectance spectra based on external parameter orthogonalization. *Geoderma,* **267,** 92-101.

Wilford, J. (2012) A weathering intensity index for the Australian continent using airborne gamma-ray spectrometry and digital terrain analysis. *Geoderma,* **183,** 124-142.

Wilford, J., de Caritat, P. & Bui, E. (2015) Modelling the abundance of soil calcium carbonate across Australia using geochemical survey data and environmental predictors. *Geoderma,* **259,** 81-92.

Wilford, J. & Thomas, M. (2013) Predicting regolith thickness in the complex weathering setting of the central Mt Lofty Ranges, South Australia. *Geoderma,* **206,** 1-13.

Wilson, M.A., Indorante, S.J., Lee, B.D., Follmer, L., Williams, D.R., Fitch, B.C., McCauley, W.M., Bathgate, J.D., Grimley, D.A. & Kleinschmidt, K. (2010) Location and expression of fragic soil properties in a loess-covered landscape, Southern Illinois, USA. *Geoderma,* **154,** 529-543.

Winowiecki, L., Vagen, T.G. & Huising, J. (2016) Effects of land cover on ecosystem services in Tanzania: A spatial assessment of soil organic carbon. *Geoderma,* **263,** 274-283.

Wu, C.F., Luo, Y.M. & Zhang, L.M. (2010) Variability of copper availability in paddy fields in relation to selected soil properties in southeast China. *Geoderma,* **156,** 200-206.

Wu, J.J., Li, Z.Y., Gao, Z.H., Wang, B.Y., Bai, L.N., Sun, B., Li, C.L. & Ding, X.Y. (2015) Degraded land detection by soil particle composition derived from multispectral remote sensing data in the Otindag Sandy Lands of China. *Geoderma,* **241,** 97-106.

Wu, X., Cai, C., Wang, J., Wei, Y. & Wang, S. (2016) Spatial variations of aggregate stability in relation to sesquioxides for zonal soils, South-central China. *Soil & Tillage Research,* **157,** 11-22.

Xiao, H., Bottcher, J. & Utermann, J. (2015) Evaluation of field-scale variability of heavy metal sorption in soils by scale factors - Scaling approach and statistical analysis. *Geoderma,* **241,** 115-125.

Xie, S.Y., Cheng, Q.M., Xing, X.T., Bao, Z.Y. & Chen, Z.J. (2010) Geochemical multifractal distribution patterns in sediments from ordered streams. *Geoderma,* **160,** 36-46.

Xu, M.X., Li, Q. & Wilson, G. (2016) Degradation of soil physicochemical quality by ephemeral gully erosion on sloping cropland of the hilly Loess Plateau, China. *Soil & Tillage Research,* **155,** 9-18.

Yang, C.F., Lu, G.N., Chen, M.Q., Xie, Y.Y., Guo, C.L., Reinfelder, J., Yi, X.Y., Wang, H. & Dang, Z. (2016) Spatial and temporal distributions of sulfur species in paddy soils affected by acid mine drainage in Dabaoshan sulfide mining area, South China. *Geoderma,* **281,** 21-29.

Yang, Y. & Wendroth, O. (2014) State-space approach to analyze field-scale bromide leaching. *Geoderma,* **217,** 161-172.

Yu, X., Wu, C., Fu, Y., Brookes, P.C. & Lu, S. (2016) Three-dimensional pore structure and carbon distribution of macroaggregates in biochar-amended soil. *European Journal of Soil Science,* **67,** 109-120.

Zadorova, T. & Penizek, V. (2011) Problems in correlation of Czech national soil classification and World Reference Base 2006. *Geoderma,* **167-68,** 54-60.

Zeng, C.Y., Yang, L., Zhu, A.X., Rossiter, D.G., Liu, J., Liu, J.Z., Qin, C.Z. & Wang, D.S. (2016) Mapping soil organic matter concentration at different scales using a mixed geographically weighted regression method. *Geoderma,* **281,** 69-82.

Zhang, S.W., Huang, Y.F., Shen, C.Y., Ye, H.C. & Du, Y.C. (2012) Spatial prediction of soil organic matter using terrain indices and categorical variables as auxiliary information. *Geoderma,* **171,** 35-43.

Zhang, Z.Q., Yu, D.S., Shi, X.Z., Weindorf, D.C., Wang, X.X. & Tan, M.Z. (2010) Effect of sampling classification patterns on SOC variability in the red soil region, China. *Soil & Tillage Research,* **110,** 2-7.

Zhu, A.X., Liu, J., Du, F., Zhang, S.J., Qin, C.Z., Burt, J., Behrens, T. & Scholten, T. (2015) Predictive soil mapping with limited sample data. *European Journal of Soil Science,* **66,** 535-547.

Zhu, A.X., Qi, F., Moore, A. & Burt, J.E. (2010) Prediction of soil properties using fuzzy membership values. *Geoderma,* **158,** 199-206.

Zhu, Q. & Lin, H. (2011) Influences of soil, terrain, and crop growth on soil moisture variation from transect to farm scales. *Geoderma,* **163,** 45-54.

Zhu, Q., Lin, H.S. & Doolittle, J.A. (2013) Functional soil mapping for site-specific soil moisture and crop yield management. *Geoderma,* **200,** 45-54.

Zhu, Y.D., Weindorf, D.C. & Zhang, W.T. (2011) Characterizing soils using a portable X-ray fluorescence spectrometer: 1. Soil texture. *Geoderma,* **167-68,** 167-177.

Zolfaghari, Z., Ayoubi, S. & Mosaddeghi, M.R. (2015) Spatial variability of some soil shrinkage indices in hilly calcareous region of western Iran. *Soil & Tillage Research,* **150,** 180-191.

Zolfaghari, Z., Mosaddeghi, M.R. & Ayoubi, S. (2016) Relationships of soil shrinkage parameters and indices with intrinsic soil properties and environmental variables in calcareous soils. *Geoderma,* **277,** 23-34.

Zuo, X.A., Zhao, X.Y., Zhao, H.L., Zhang, T.H., Li, Y.L., Wang, S.K., Li, W.J. & Powers, R. (2012) Scale dependent effects of environmental factors on vegetation pattern and composition in Horqin Sandy Land, Northern China. *Geoderma,* **173,** 1-9.
