# Supplementary material for: Generalizing soil properties in geographic space: Approaches used and ways forward
Source: PLoS One. 2018 Dec 21;13(12):e0208823. doi: 10.1371/journal.pone.0208823 (PMC6303050; doi:10.1371/journal.pone.0208823)
Supplement: S1 Table — In bold are evidenced the best validation values. ANN = Artificial Neural Network; OK = Ordinary Kriging; UK = Universal Kriging; IDW = Inverse Distance Weighted; SLR = Stepwise Linear Regression; RF = Random Forest; RK = Regression Kriging; BK = Block Kriging; RST = Regularized Spline with Tension; OCK = Ordinary Co-Kriging; HASM = High accuracy surface modelling; HASM_LU = High Accuracy Surface Modelling with Land Use information; OK_LU = Ordinary Kriging with Land Use information; SK = Stratified Kriging; RK_GLM = Regression-Kriging using a Generalized Linear Model; RK/SMLR = regression kriging using stepwise multiple linear regression; RK/RT = regression kriging using regression trees to map the global spatial trend; DS = Disaggregation simulation; AWM = Area-weighted mean; KED = Kriging with external drift; KST = Kriging combined with Soil-Type information; KLU = Kriging combined with Land Use; KLUST = Kriging combined with Soil Type; RK(TOPO, TOPOVI, TOPOSOIL and TOPOVISOIL) = Regression Kriging with four auxiliary data sets; GWR = Geographically Weighted Regression. CKmc = multi-collocated CoKriging. RMSE = Root Mean Square Error; Var = Variance explained (%); R2 = Coefficient of determination; MAE = Mean Absolute Error; RPD = Residual Prediction. (DOCX) [file pone.0208823.s002.docx]

**S1 Table** **– List of the 12 papers used in validation of comparison between the different method techniques**. In bold are evidenced the best validation values. ANN=Artificial Neural Network; OK=Ordinary Kriging; UK=Universal Kriging; IDW=Inverse Distance Weighted; SLR=Stepwise Linear Regression; RF=Random Forest; RK=Regression Kriging; BK=Block Kriging; RST=Regularized Spline with Tension; OCK=Ordinary Co-Kriging; HASM= High accuracy surface modelling; HASM_LU= High Accuracy Surface Modelling with Land Use information; OK_LU= Ordinary Kriging with Land Use information; SK=Stratified Kriging; RK_GLM= Regression-Kriging using a Generalized Linear Model; RK/SMLR = regression kriging using stepwise multiple linear regression; RK/RT = regression kriging using regression trees to map the global spatial trend; DS=Disaggregation simulation ; AWM=Area-weighted mean; KED=Kriging with external drift; KST =Kriging combined with Soil-Type information; KLU= Kriging combined with Land Use; KLUST=Kriging combined with Soil Type; RK(TOPO, TOPOVI, TOPOSOIL and TOPOVISOIL)=Regression Kriging with four auxiliary data sets; GWR=Geographically Weighted Regression. CKmc=multi-collocated CoKriging. RMSE=Root Mean Square Error; Var=Variance explained (%); R^2^= Coefficient of determination; MAE = Mean Absolute Error; RPD=Residual Prediction.

| **Study** | **Predicted**  **variable** | **N. samples** | **Country** | **Altitudinal range (m)** | **Extent (km2)** | **Density (n. samples/km2)** | **Validation measure** | **Model technique and validation values** | |
| --- | --- | --- | --- | --- | --- | --- | --- | --- | --- |
| **Cambule et al., 2013** | SOC | 50 | Mozambique |  | 10400 | 0.0048 | RMSE | OK1=0.50 ; OK2=0.36  KED1=**0.42**; KED2=**0.31**  LR1=0.45; LR2=**0.31** | |
| **Castaldi et al., 2014** | clay, sand, SOM | 72 | Italy | 8-8 | 2.9 | 24.82 | RMSE | OK = 4.46, 5.18, 0.33  PLSR = 5.95, 6.69, 0.41  OLS PLSR = 5.45, 5.96, 0.40  OLS PCA = 4.97, 5.42, **0.27**  OLS MNF(minumum noise fraction) =5.76, 6.22, 0.15  Linear mixed effect models (LMEM) MNF = **4.07**, **4.90** (Hybrid) | |
| **de Carvalho et al., 2014** | Clay, sand, SOC, pH at 6 depths | 208 | Brazil |  | 390 | 0.53 | R^2^ | *Clay*  OK=**0.19, 0.19**  RK=0.18, 0.18, 0.15  LM=0.09 | *Sand*  OK=0.17, 0.15, 0.04  RK=**0.17, 0.2, 0.27** |
| **Guo et al., 2015** | SOM | 2511 | China | 0.5-659.2 | 996 | 2.52 | Var | LR= 43.8 (Var)  RF=66.67  RF-RK=**88.65** | |
| **Hoffmann et al., 2014** | SOC | 409 | Switzerland | 900-2400 | 8.6 | 47.56 | R^2^ | IDW =0.323  OK=**0.377**  BK (250m)=0.353; BK (500m) = 0.319  RK = 0.338 | |
| **Li, 2010** | SOM | 335 | China | 432-929 | 430 | 0.78 | R^2^ | OK =**0.56**  UK = 0.55  RK(TOPOVI) = 0.55; RK(TOPOSOIL)=0.55; RK(TOPOVISOIL)=0.48 | |
| **Liu et al., 2015** | SOM | 294 | China | 892-2844 |  |  | RMSE | IDW=**13**  UK=13.06 | |
| **Kerry et al., 2012** | SOC | 6862 | Ireland | 0-225 |  |  | MAE | Kriging = 7.44  Regression kriging =**4.62** | |
| **Phachomphon et al., 2010** | SOC | 2806 | Laos | 662-2806 | 230000 | 0.0122 | MAE | OK=3.3  IDW=2.2  RST=3.0  OCK=**2.1** | |
| **Shi et al., 2011** | pH, alkali-hydrolysable nitrogen, TC, N, K, Al, Ca, Mg, Zn | 150 | China |  | 6156.92 | 0.024 | RMSE | HASM_LU =**0.19, 36.69, 5.07, 0.31, 5.94, 13.71, 2.6, 2.23,** 36.87  HASM = **0.19**, 48.95, 5.88, 0.43, 6.61, 14.93, 3.6, 2.44, **34.62**  OK_LU=0.2, 37.28, 5.1, 0.32 ,6.31, 13.77, 2.61, **2.23**, 37.14  SK= 0.25, 51.92, 6.67 ,0.39, 7.35 ,14.19, 3.73 ,2.58 ,55.25  RK_GLM= 0.26, 61.49, 6.34, 0.42, 7.40, 17.06, 4.08 ,2.42, 35.15 | |
| **Vaysse, 2015** | clay, silt, sand, coarse fragment, OC, pH, CEC | 2014 | France |  | 27236 | 0.074 | RMSE | *Clay*  0–5= 104.72, 120.79, 99.97, **84.73**  5–15= 97.97, 112.36, 99.70, **84.28**  15–30 =100.65, 117.08, 100.34, **88.20**  30–60 = 90.67, 117.05, 94.49, **88.47**  *Silt*  0–5 =114.44, 120.53, **97.56**, 97.59  5–15= 109.77, 117.49, 98.19, **98.17**  15–30= 106.37, 111.58, **93.58**, 93.85  30–60 =119.03, 116.05, **97.11**, 97.83  *Sand*  0–5 =159.75, 176.74, **139.80**, 140.03  5–15 =158.57, 171.95, **140.23**, 140.50  15–30 =159.31, 176.73, 140.16, **139.77**  30–60 =156.50, 171.64, **146.57**, 148.82  *Coarse fragment*  0–5= 17.67, 16.53, 9.40, **9.03**  5–15= 17.92, 16.80, 9.28, **8.89**  15–30=24.08, 20.52, 10.22, **8.37**  30–60 = 20.06, 23.32, 14.53, **8.98** | *Organic carbon*  0–5 =108.24, 93.20, **18.31**, 18.64  5–15 =72.90, 75.13, **18.47**, 19.15  15–30 =74.94, 66.52, 19.64, **18.61**  30–60 =120.20, 73.86, **13.12**, 13.41  *pH*  0–5 =0.91, 0.84, **0.78, 0.78**  5–15 =0.91, 0.84, **0.78 ,0.78**  15–30 =0.90, 0.82, 0.76**, 0.75**  30–60 =**0.74**, 0.86, 0.82, 0.83  *CEC*  0–5= 11.74, 10.36, **9.49**, 9.50  5–15 =10.80, 9.97, **9.46**, 9.58  15–30 =10.32, 9.69, **9.16**, 9.24  30–60 =10.38, 7.86 ,**6.62**, 6.80  The methods used are: DS, AWM, RF, KED |
| **Wang et al., 2015** | TC, TN | 675 | US |  | 25134 | 0.07 | R^2^ | RF=0.83, 0.91 (R^2^)  PSR (penalized spline regression)=**0.93**, 0.91 | |
| **Wang K., 2013** | TN | 353 | China | 140-1200 | 1260.00 | 0.28 | R^2^_adjusted | OCK = **0.6858**  GWR = 0.5746 | |
| **Zhang et al., 2010** | SOM | 469 | China | 0-1705 | 2229.5 | 0.21 | RMSE | OK=1.33  MLR(Multiple Linear regression)=1.26  RK=**0.83** | |

**References**

Cambule, A.H., Rossiter, D.G., Stoorvogel, J.J., 2013. A methodology for digital soil mapping in poorly-accessible areas. Geoderma 192, 341-353.

Castaldi, F., Casa, R., Castrignano, A., Pascucci, S., Palombo, A., Pignatti, S., 2014. Estimation of soil properties at the field scale from satellite data: a comparison between spatial and non-spatial techniques. Eur J Soil Sci 65(6), 842-851.

de Carvalho, W., Lagacherie, P., Chagas, C.D., Calderano, B., Bhering, S.B., 2014. A regional-scale assessment of digital mapping of soil attributes in a tropical hillslope environment. Geoderma 232, 479-486.

Guo, P.T., Li, M.F., Luo, W., Tang, Q.F., Liu, Z.W., Lin, Z.M., 2015. Digital mapping of soil organic matter for rubber plantation at regional scale: An application of random forest plus residuals kriging approach. Geoderma 237, 49-59.

Hoffmann, U., Hoffmann, T., Jurasinski, G., Glatzel, S., Kuhn, N.J., 2014. Assessing the spatial variability of soil organic carbon stocks in an alpine setting (Grindelwald, Swiss Alps). Geoderma 232, 270-283.

Kerry, R., Goovaerts, P., Rawlins, B.G., Marchant, B.P., 2012. Disaggregation of legacy soil data using area to point kriging for mapping soil organic carbon at the regional scale. Geoderma 170, 347-358.

Li, Y., 2010. Can the spatial prediction of soil organic matter contents at various sampling scales be improved by using regression kriging with auxiliary information? Geoderma 159(1-2), 63-75.

Liu, S.L., An, N.N., Yang, J.J., Dong, S.K., Wang, C., Yin, Y.J., 2015. Prediction of soil organic matter variability associated with different land use types in mountainous landscape in southwestern Yunnan province, China. Catena 133, 137-144.

Phachomphon, K., Dlamini, P., Chaplot, V., 2010. Estimating carbon stocks at a regional level using soil information and easily accessible auxiliary variables. Geoderma 155(3-4), 372-380.

Shi, W.J., Liu, J.Y., Du, Z.P., Stein, A., Yue, T.X., 2011. Surface modelling of soil properties based on land use information. Geoderma 162(3-4), 347-357.

Vaysse, K., & Lagacherie, P. , 2015. Evaluating digital soil mapping approaches for mapping GlobalSoilMap soil properties from legacy data in Languedoc-Roussillon (France). Geoderma Regional 4.

Wang, D.D., Chakraborty, S., Weindorf, D.C., Li, B., Sharma, A., Paul, S., Ali, M.N., 2015. Synthesized use of VisNIR DRS and PXRF for soil characterization: Total carbon and total nitrogen. Geoderma 243, 157-167.

Wang K., Z.C., & Li W. , 2013. Predictive mapping of soil total nitrogen at a regional scale: a comparison between geographically weighted regression and cokriging. Applied Geography 42.

Zhang, Z.Q., Yu, D.S., Shi, X.Z., Warner, E., Ren, H.Y., Sun, W.X., Tan, M.Z., Wang, H.J., 2010. Application of categorical information in the spatial prediction of soil organic carbon in the red soil area of China. Soil Sci Plant Nutr 56(2), 307-318.
