# Supplementary material for: Generalizing soil properties in geographic space: Approaches used and ways forward
Source: PLoS One. 2018 Dec 21;13(12):e0208823. doi: 10.1371/journal.pone.0208823 (PMC6303050; doi:10.1371/journal.pone.0208823)
Supplement: S2 Table — (DOCX) [file pone.0208823.s003.docx]

**S2 Table – List of the 92 selected papers using only one type of approach to spatialize soil properties.**

| **Study** | **Soil properties** | **N. samples** | **Country** | **Altitude (m)** | **Extent of study area (Km2)** | **Density (N. samples/km2)** | **Method** |
| --- | --- | --- | --- | --- | --- | --- | --- |
| **(Bogunovic et al., 2014)** | P_avail, K_avail, pH, SOM | 330 | Croatia | 112 | 0.84 | 392.85 | IDW, Sp, OK |
| **Camargo et al., 2015** | Clay, iron, Phosphate |  | Brazil |  |  |  | Kriging |
| **Cruz-Cardenas et al., 2014** | Ca, electrical conductivity (EC), SOC, K, Mg, SOM, Na, pH, sodium absorption ratio (SAR) | 4440 | Mexico |  | Whole Mexico | 0.0022 | Ordinary kriging, simple kriging |
| **Dou et al., 2010** | SOC | 48 | Alaska |  | 1800 (km) | 0.026 | OK |
| **Horta et al. 2010** | SOC |  | Portugal |  | 2900 |  | Direct Sequential Co-Simulation (Co-DSS) |
| **Liu et al., 2013** | TN, TP | 382 | China | 100-3000 | 620000 | 0.00062 | OK |
| **Mansuy et al., 2014** | SOC, TN, C:N, clay, silt, sand, bulk density | 538 | Canada |  | 2900000 | 0.00018 | k nearest-neighbours |
| **Szopka et al. 2012** | Pb | 372 | Poland | 500-1350 | 440 | 0.84 | Ordinary kriging |
| **Tanikawa et al., 2013** | TS, TC, Bulk density, Al, Fe, Si | 50 | Japan | 210-270 | 0.021 | 2380.9 | Kriging |
| **Ballabio et al., 2012** | SOC | 83 | Italy | 1900-2000 | 2 | 41.5 | RK |
| **Colombo et al., 2015** | Nitrate | 134 | Italy | 0-222 | 107 | 1.25 | Co-kriging |
| **De Benedetto et al., 2012** | Clay | 22 | Italy |  | 0.01 | 2210 | KED |
| **Lagacherie et al., 2013** | Clay | 200 | France |  | 24.6 |  | Co-kriging, Block-co-kriging |
| **Levi and Rasmussen, 2014** | Clay, sand, silt, gravel, cobble, total coarse fragments, redness rating, loss on ignition | 52 | US | 1273-1655 | 62.65 | 0.830007981 | RK |
| **Li, 2010** | SOM | 335 | China | 432-929 | 400 | 0.84 | RK |
| **Orton et al., 2014** | clay | 50 | Australia | 250-700 | 1025 | 0.049 | ATP kriging |
| **Roger et al., 2014** | TP, P_avail | 250 | Switzerland |  | 1670.7 | 0.15 | RK |
| **Sun et al., 2012** | pH, clay, TC | 985 | Australia |  | 38 | 25.92 | RK |
| **Vasques et al., 2010** | TC | 141 | US |  | 3585 | 0.039 | BK, RK |
| **Araujo et al., 2015** | SOC, P, CEC, EC, Ca, Mg, K, Al, acidity, pH, BS (base saturation) | 198 | Brazil |  |  |  | PLSR |
| **Araujo et al., 2014** | SOM, clay | 7172 | Brazil |  | 2078180 | 0.0034 | PLSR, boosted regression trees, support vector machine |
| **Ballabio et al., 2016** | Sand, silt, clay | 19969 | Europe |  | Whole Europe |  | MARS |
| **Behrens et al., 2010** | Type/class |  | Germany |  | 300 |  | CT |
| **Cavazzi et al., 2013** | Type/class |  | Ireland |  | 1081 |  | ANN, RF |
| **Coulouma et al., 2016** | Clay | 242 | France |  | 0.1 | 2420 | Regression |
| **D'Acqui et al., 2010** | SOC, IC, CEC, clay, pH | 198 | Italy |  | 24333 | 0.0081 | PLSR |
| **Debaene et al., 2014** | pH, P_2_O_5,_ K_2_O, Mg SOC, Sand, silt, clay | 403 | Poland |  | 0.53 | 760.4 | PSLR |
| **Dieleman et al., 2013** | SOC | 105 | Papua New Guinea | 100-3050 | 760 | 0.14 | LR |
| **Doetterl et al., 2013** | CS | 50 | Luxembourg |  | 500 | 0.1 | LMM |
| **Goge et al., 2014** | CaCO3 | 144 | France |  | 24 | 0.15 | PLSR, local regression |
| **Gomez et al., 2012** | Clay, sand, silt, calcium carbonate, iron, CEC, SOC, pH | 95 | France |  | 24.6 | 3.8 | PLSR |
| **Heung et al., 2014** | Parental material |  | Canada | 0-2555 | 5472 |  | RF |
| **Hu et al., 2014** | Clay, silt, sand, TN, total phosphorus, bulk density, SOM | 124 | China | 1056 - 1130 | 0.2 | 620 | Ordinary Least squared (OLS), generalized least squared (GLS) |
| **Jafari et al., 2014** | Type/class | 126 | Iran | 1600-2100 | 900 | 0.14 | BRT |
| **Ji et al. 2016** | pH, SOM, TN | 225 | China |  | 50000 | 0.045 | Linear Weighted regression |
| **Ji et al. 2015** | SOM | 104 | China |  |  |  | PSLR |
| **Kempen et al., 2012** | Soil type | 125 | Netherland |  | 167.5 | 0.74 | generalised linear geostatistical model (GLGM) |
| **Kim and Zheng, 2011** | K, pH, TP, nitrate | 193 | Korea |  | 4 | 48.5 | OLSR |
| **Kim et al., 2012** | Type/class | 108 | US |  | 418 | 0.258373206 | CT |
| **Kodaira and Shibusawa, 2013** | moisture content (MC), soil organic matter (SOM), pH, electrical conductivity (EC), cation exchange capacity (CEC), total carbon (C-t), ammonium nitrogen (N-a), hot water extractable nitrogen (N-h), nitrate nitrogen (N-n), total nitrogen (N-t), available phosphorus (P-a), and phosphorus absorptive coefficient (PAC). | 144 | Japan | 0 | 0.31 | 464.5 | PLSR |
| **Kovacevic et al., 2010** | SOM, pH, N, potassium oxide, phosphorus pentoxide, sulphur, CEC, sand, clay, silt, type/class | 151 | Serbia | 106-290 | 12.72 | 11.87 | SVM, LR |
| **Kumar et al., 2012** | SOC | 878 | US | 6-1050 | 117599 | 0.0074 | GWRK |
| **Lacarce et al., 2012** | Pb | 2091 | France |  | 550000 | 0.0038 | LMM (linear mixed model) |
| **Lacoste et al., 2014** | SOC, bulk | 70 | France |  | 10 | 7 | MLR |
| **Lagacherie et al., 2013** | Clay, sand, CEC | 205 | Tunisia | 0-226 | 300 | 0.68 | PLSR |
| **Láng et al. 2016** | Soil group | 2136 | Africa |  | Whole Africa |  | Random Forest |
| **Lemercier et al., 2012** | Parental material, soil drainage | 1148 | France |  | 4645 | 0.25 | BCT, RT |
| **Liess et al., 2012** | Sand, clay, silt | 56 | Ecuador | 1720-3160 | 30 | 1.8 | RT, RF |
| **Liu et al., 2012** | Clay, sand | 51 | Canada |  | 430 | 0.12 | MLR |
| **Maynard and Johnson, 2014** | Clay, sum of bases, SOM | 35 | US | 100-700 | 23 | 1.5 | OLSR |
| **Marchant et al., 2011** | Cd | 2200 | France |  | Whole France 550000 | 0.004 | LMM |
| **McDowell et al., 2012** | TC | 305 | US |  | 16078 | 0.018970021 | PLSR, RF, RT |
| **Miller et al., 2015** | SOC, silt, bulk, topsoil thickness | 157 | Germany |  |  |  | MLR |
| **Mouazen et al. 2010** | organic carbon (OC) and extractable forms of potassium (K), sodium (Na), magnesium (Mg) and phosphorous (P) | 168 | Belgium |  |  |  | principal component regression (PCR), partial least squares regression (PLSR) and back propagation neural network (BPNN) |
| **Munoz and Kravchenko, 2011** | TC | 50 | US |  | 0.24 | 208.3 | MLR, PLSR |
| **Nawar et al., 2016** | SOM, clay | 102 | Egypt |  | 175 | 0.58 | PLSR, SVR (SUPPORT VECTOR REGRESSION), MARS |
| **Nocita et al., 2013** | SOC | 107 | Luxembourg | 450 | 420 | 0.25 | PLSR |
| **Odgers et al., 2014** | Type/class | 1080 | Australia |  | 68000 | 0.016 | DSMART |
| **Orton et al., 2014** | SOC | 179 | Australia |  |  |  | LMC |
| **Rad et al., 2014** | Type/class | 150 | Iran | 18-158 | 850 | 0.18 | RF |
| **Parry and Charman, 2013** | SOC | 29 | UK |  | 471 | 0.06 | LR |
| **Pascucci et al., 2014** | SOC | 53 | Italy | 30-30 | 0.09 | 588.8 | PLSR, cubist regression |
| **Poggio et al., 2010** | Available water capacity |  | Scotland |  |  |  | GAM |
| **Poggio et al., 2013** | SOM | 7807 | Scotland |  | 78000 |  | GAM, RT |
| **Poggio and Gimona, 2014** | SOC |  | Scotland |  | Whole scotland78000 | 0.1 | GAM_residuals |
| **Poggio et al. 2016** | SOM |  | Scotland |  | 12100 |  | Bayesian |
| **Priori et al., 2014** | Sand, clay, stoniness | 70 | Italy |  | 0.31 | 228.0130293 | SVM, ANN |
| **Qin et al., 2012** | SOM | 48 | China | 224-393 | 4 | 12 | MLR |
| **Rossel et al., 2011** | Water content, bulk density, clay, silt, sand, total C, Total K, Total N, C :N, total p, available P, pH, CEC, Ca^2+^, K^+^, Mg^2+^, Na^+^, Fe | 21492 | Australia |  | 7692000 |  | Model tree |
| **Sequeira et al., 2014** | Bulk density | 2680 | US |  | 9857306 | 0.00027 | RF |
| **Shi et al., 2011** | SOM | 2732 | China | 0-5000 | 9596961 | 0.0028 | PSLR |
| **Silveira et al., 2013** | Type/class |  | Brazil |  |  |  | ANN |
| **Stevens et al., 2014** | SOC | 2449 | Belgium |  | 9921 | 0.25 | MLR |
| **Sun et al., 2012** | SOM | 1231 | China | 0-607 | 102600 | 0.01 | Bayesian Maximum Entropy (BME) |
| **Suuster et al., 2012** | SOC | 8697 | Estonia |  |  |  | i) robust prediction by using medians, (ii) ANCOVA, (iii) mixed-model analysis and (iv) random forests |
| **Taghizadeh-Mehrjardi et al., 2014** | Salinity | 173 | Iran | 944-1944 | 720 | 0.24 | RT |
| **Taalab et al. 2015** | Bulk density | 164 | Ireland | 0-900 | 6062 | 0.027 | RF, Bayesian network naive, Bayesian network hierarchical |
| **Taylor et al., 2013** | Soil and watertable depth | 41 | France | 20-230 | 65 | 0.64 | RT |
| Vašát **et al. 2014** | pH, Ca, Cu, Fe, K, Mg, Mn, P and Zn | 97 | Czech Republic |  | 0.06 | 1616.6 | MLR, PLSR |
| **Vågen et al. 2016** | SOC, pH, sand, sum of exchangeable bases | 10473 | Africa |  | Whole Africa |  | Random Forest |
| **Vendrame et al. 2012** | Clay, silt, sand, pH, totalC, P, Ca^2+^, Mg^2+^, K^+^, Al^3+^, CEC, exchangeable bases (EB), base saturation (BS) | 148 | Brazil |  |  |  | PLSR |
| **Rossel and Behrens, 2010** | SOC, clay, pH | 1104 | Australia |  |  |  | MLR, PLSR, MARS, SVM, RF, BRT, ANN |
| **Vohland et al., 2011** | SOC | 109 | Germany |  |  |  | PLSR, a combination of PLSR with a genetic algorithm (GA-PLSR), support vector machine regression (SVMR |
| **Vohland et al., 2014** | SOC, TN, pH, MINC | 60 | Germany |  | 150 |  | PLSR, CARS_PLS |
| **Watt and Palmer, 2012** | C:N | 1573 | New Zealand |  | 268021 | 0.0058 | RK |
| **Wetterlind et al., 2015** | Clay, silt, sand, SOM | 40 | Sweden | 0 | 0.92 | 43.47 | Linear regression, PLSR |
| **Wilford et al., 2015** | Calcium carbonate | 1311 | Australia |  | Whole Australia | 0.00172 | machine learning decision tree |
| **Winowiecki et al., 2016** | SOC | 2052 | Tanzania | 281-2090 | 945090 | 0.0022 | Linear mixed effects models |
| **Wijewardane et al., 2016** | OC, IC, TC, Sand ,Clay, pH, P, CEC, | 185 | US |  |  |  | PLSR, RF, ANN, SVM |
| **Zhu et al. 2010** | Sand, silt, clay, horizons depths |  | US |  |  |  | MLR |
| **Zhu et al. 2015** | SOM | 22 | China | 276-363 | 60 | 0.36 | MLR |

**References**

Araujo, S.R., Soderstrom, M., Eriksson, J., Isendahl, C., Stenborg, P., Dematte, J.A.M., 2015. Determining soil properties in Amazonian Dark Earths by reflectance spectroscopy. Geoderma 237, 308-317.

Araujo, S.R., Wetterlind, J., Dematte, J.A.M., Stenberg, B., 2014. Improving the prediction performance of a large tropical vis-NIR spectroscopic soil library from Brazil by clustering into smaller subsets or use of data mining calibration techniques. Eur J Soil Sci 65(5), 718-729.

Ballabio, C., Fava, F., Rosenmund, A., 2012. A plant ecology approach to digital soil mapping, improving the prediction of soil organic carbon content in alpine grasslands. Geoderma 187, 102-116.

Ballabio, C., Panagos, P., Monatanarella, L., 2016. Mapping topsoil physical properties at European scale using the LUCAS database. Geoderma 261, 110-123.

Behrens, T., Zhu, A.X., Schmidt, K., Scholten, T., 2010. Multi-scale digital terrain analysis and feature selection for digital soil mapping. Geoderma 155(3-4), 175-185.

Bogunovic, I., Mesic, M., Zgorelec, Z., Jurisic, A., Bilandzija, D., 2014. Spatial variation of soil nutrients on sandy-loam soil. Soil Till Res 144, 174-183.

Camargo, L.A., Marques, J., Barron, V., Alleoni, L.R.F., Barbosa, R.S., Pereira, G.T., 2015. Mapping of clay, iron oxide and adsorbed phosphate in Oxisols using diffuse reflectance spectroscopy. Geoderma 251, 124-132.

Cavazzi, S., Corstanje, R., Mayr, T., Hannam, J., Fealy, R., 2013. Are fine resolution digital elevation models always the best choice in digital soil mapping? Geoderma 195, 111-121.

Colombo, C., Palumbo, G., Sellitto, V.M., Di Iorio, E., Castrignano, A., Stelluti, M., 2015. The effects of land use and landscape on soil nitrate availability in Southern Italy (Molise region). Geoderma 239, 1-12.

Coulouma, G., Caner, L., Loonstra, E.H., Lagacherie, P., 2016. Analysing the proximal gamma radiometry in contrasting Mediterranean landscapes: Towards a regional prediction of clay content. Geoderma 266, 127-135.

Cruz-Cardenas, G., Lopez-Mata, L., Ortiz-Solorio, C.A., Villasenor, J.L., Ortiz, E., Silva, J.T., Estrada-Godoy, F., 2014. Interpolation of Mexican soil properties at a scale of 1:1,000,000. Geoderma 213, 29-35.

D'Acqui, L.P., Pucci, A., Janik, L.J., 2010. Soil properties prediction of western Mediterranean islands with similar climatic environments by means of mid-infrared diffuse reflectance spectroscopy. Eur J Soil Sci 61(6), 865-876.

De Benedetto, D., Castrignano, A., Sollitto, D., Modugno, F., Buttafuoco, G., lo Papa, G., 2012. Integrating geophysical and geostatistical techniques to map the spatial variation of clay. Geoderma 171, 53-63.

Debaene, G., Niedzwiecki, J., Pecio, A., Zurek, A., 2014. Effect of the number of calibration samples on the prediction of several soil properties at the farm-scale. Geoderma 214, 114-125.

Dieleman, W.I.J., Venter, M., Ramachandra, A., Krockenberger, A.K., Bird, M.I., 2013. Soil carbon stocks vary predictably with altitude in tropical forests: Implications for soil carbon storage. Geoderma 204, 59-67.

Doetterl, S., Stevens, A., van Oost, K., Quine, T.A., van Wesemael, B., 2013. Spatially-explicit regional-scale prediction of soil organic carbon stocks in cropland using environmental variables and mixed model approaches. Geoderma 204, 31-42.

Dou, F.G., Yu, X., Ping, C.L., Michaelson, G., Guo, L.D., Jorgenson, T., 2010. Spatial variation of tundra soil organic carbon along the coastline of northern Alaska. Geoderma 154(3-4), 328-335.

Goge, F., Gomez, C., Jolivet, C., Joffre, R., 2014. Which strategy is best to predict soil properties of a local site from a national Vis-NIR database? Geoderma 213, 1-9.

Gomez, C., Lagacherie, P., Coulouma, G., 2012. Regional predictions of eight common soil properties and their spatial structures from hyperspectral Vis-NIR data. Geoderma 189, 176-185.

Heung, B., Bulmer, C.E., Schmidt, M.G., 2014. Predictive soil parent material mapping at a regional-scale: A Random Forest approach. Geoderma 214, 141-154.

Hu, P., Wang, D., Cassidy, M.J., Stanier, S.A., 2014. Predicting the resistance profile of a spudcan penetrating sand overlying clay. Can Geotech J 51(10), 1151-1164.

Jafari, A., Khademi, H., Finke, P.A., Van de Wauw, J., Ayoubi, S., 2014. Spatial prediction of soil great groups by boosted regression trees using a limited point dataset in an arid region, southeastern Iran. Geoderma 232, 148-163.

Kempen, B., Brus, D.J., Heuvelink, G.B.M., 2012. Soil type mapping using the generalised linear geostatistical model: A case study in a Dutch cultivated peatland. Geoderma 189, 540-553.

Kim, D., Zheng, Y.B., 2011. Scale-dependent predictability of DEM-based landform attributes for soil spatial variability in a coastal dune system. Geoderma 164(3-4), 181-194.

Kim, J., Grunwald, S., Rivero, R.G., Robbins, R., 2012. Multi-scale Modeling of Soil Series Using Remote Sensing in a Wetland Ecosystem. Soil Sci Soc Am J 76(6), 2327-2341.

Kodaira, M., Shibusawa, S., 2013. Using a mobile real-time soil visible-near infrared sensor for high resolution soil property mapping. Geoderma 199, 64-79.

Kovacevic, M., Bajat, B., Gajic, B., 2010. Soil type classification and estimation of soil properties using support vector machines. Geoderma 154(3-4), 340-347.

Kumar, S., Lal, R., Liu, D.S., 2012. A geographically weighted regression kriging approach for mapping soil organic carbon stock. Geoderma 189, 627-634.

Lacarce, E., Saby, N.P.A., Martin, M.P., Marchant, B.P., Boulonne, L., Meersmans, J., Jolivet, C., Bispo, A., Arrouays, D., 2012. Mapping soil Pb stocks and availability in mainland France combining regression trees with robust geostatistics. Geoderma 170, 359-368.

Lacoste, M., Minasny, B., McBratney, A., Michot, D., Viaud, V., Walter, C., 2014. High resolution 3D mapping of soil organic carbon in a heterogeneous agricultural landscape. Geoderma 213, 296-311.

Lagacherie, P., Sneep, A.R., Gomez, C., Bacha, S., Coulouma, G., Hamrouni, M.H., Mekki, I., 2013. Combining Vis-NIR hyperspectral imagery and legacy measured soil profiles to map subsurface soil properties in a Mediterranean area (Cap-Bon, Tunisia). Geoderma 209, 168-176.

Lemercier, B., Lacoste, M., Loum, M., Walter, C., 2012. Extrapolation at regional scale of local soil knowledge using boosted classification trees: A two-step approach. Geoderma 171, 75-84.

Levi, M.R., Rasmussen, C., 2014. Covariate selection with iterative principal component analysis for predicting physical soil properties. Geoderma 219, 46-57.

Liess, M., Glaser, B., Huwe, B., 2012. Uncertainty in the spatial prediction of soil texture Comparison of regression tree and Random Forest models. Geoderma 170, 70-79.

Liu, F., Geng, X.Y., Zhu, A.X., Fraser, W., Waddell, A., 2012. Soil texture mapping over low relief areas using land surface feedback dynamic patterns extracted from MODIS. Geoderma 171, 44-52.

Liu, Z.P., Shao, M.A., Wang, Y.Q., 2013. Spatial patterns of soil total nitrogen and soil total phosphorus across the entire Loess Plateau region of China. Geoderma 197, 67-78.

Mansuy, N., Thiffault, E., Pare, D., Bernier, P., Guindon, L., Villemaire, P., Poirier, V., Beaudoin, A., 2014. Digital mapping of soil properties in Canadian managed forests at 250 m of resolution using the k-nearest neighbor method. Geoderma 235, 59-73.

Marchant, B.P., Saby, N.P.A., Jolivet, C.C., Arrouays, D., Lark, R.M., 2011. Spatial prediction of soil properties with copulas. Geoderma 162(3-4), 327-334.

Maynard, J.J., Johnson, M.G., 2014. Scale-dependency of LiDAR derived terrain attributes in quantitative soil-landscape modeling: Effects of grid resolution vs. neighborhood extent. Geoderma 230, 29-40.

McDowell, M.L., Bruland, G.L., Deenik, J.L., Grunwald, S., Knox, N.M., 2012. Soil total carbon analysis in Hawaiian soils with visible, near-infrared and mid-infrared diffuse reflectance spectroscopy. Geoderma 189, 312-320.

Miller, B.A., Koszinski, S., Wehrhan, M., Sommer, M., 2015. Impact of multi-scale predictor selection for modeling soil properties. Geoderma 239, 97-106.

Munoz, J.D., Kravchenko, A., 2011. Soil carbon mapping using on-the-go near infrared spectroscopy, topography and aerial photographs. Geoderma 166(1), 102-110.

Nawar, S., Buddenbaum, H., Hill, J., Kozak, J., Mouazen, A.M., 2016. Estimating the soil clay content and organic matter by means of different calibration methods of vis-NIR diffuse reflectance spectroscopy. Soil Till Res 155, 510-522.

Nocita, M., Stevens, A., Noon, C., van Wesemael, B., 2013. Prediction of soil organic carbon for different levels of soil moisture using Vis-NIR spectroscopy. Geoderma 199, 37-42.

Odgers, N.P., Sun, W., McBratney, A.B., Minasny, B., Clifford, D., 2014. Disaggregating and harmonising soil map units through resampled classification trees. Geoderma 214, 91-100.

Orton, T.G., Pringle, M.J., Page, K.L., Dalal, R.C., Bishop, T.F.A., 2014. Spatial prediction of soil organic carbon stock using a linear model of coregionalisation. Geoderma 230, 119-130.

Parry, L.E., Charman, D.J., 2013. Modelling soil organic carbon distribution in blanket peatlands at a landscape scale. Geoderma 211, 75-84.

Pascucci, S., Casa, R., Belviso, C., Palombo, A., Pignatti, S., Castaldi, F., 2014. Estimation of soil organic carbon from airborne hyperspectral thermal infrared data: a case study. Eur J Soil Sci 65(6), 865-875.

Poggio, L., Gimona, A., 2014. National scale 3D modelling of soil organic carbon stocks with uncertainty propagation - An example from Scotland. Geoderma 232, 284-299.

Poggio, L., Gimona, A., Brewer, M.J., 2013. Regional scale mapping of soil properties and their uncertainty with a large number of satellite-derived covariates. Geoderma 209, 1-14.

Poggio, L., Gimona, A., Brown, I., Castellazzi, M., 2010. Soil available water capacity interpolation and spatial uncertainty modelling at multiple geographical extents. Geoderma 160(2), 175-188.

Priori, S., Bianconi, N., Costantini, E.A.C., 2014. Can gamma-radiometrics predict soil textural data and stoniness in different parent materials? A comparison of two machine-learning methods. Geoderma 226, 354-364.

Qin, C.Z., Zhu, A.X., Qiu, W.L., Lu, Y.J., Li, B.L., Pei, T., 2012. Mapping soil organic matter in small low-relief catchments using fuzzy slope position information. Geoderma 171, 64-74.

Rad, M.R.P., Toomanian, N., Khormali, F., Brungard, C.W., Komaki, C.B., Bogaert, P., 2014. Updating soil survey maps using random forest and conditioned Latin hypercube sampling in the loess derived soils of northern Iran. Geoderma 232, 97-106.

Roger, A., Libohova, Z., Rossier, N., Joost, S., Maltas, A., Frossard, E., Sinaj, S., 2014. Spatial variability of soil phosphorus in the Fribourg canton, Switzerland. Geoderma 217, 26-36.

Rossel, R.A.V., Behrens, T., 2010. Using data mining to model and interpret soil diffuse reflectance spectra. Geoderma 158(1-2), 46-54.

Rossel, R.A.V., Chappell, A., de Caritat, P., McKenzie, N.J., 2011. On the soil information content of visible-near infrared reflectance spectra. Eur J Soil Sci 62(3), 442-453.

Sequeira, C.H., Wills, S.A., Seybold, C.A., West, L.T., 2014. Predicting soil bulk density for incomplete databases. Geoderma 213, 64-73.

Silveira, C.T., Oka-Fiori, C., Santos, L.J.C., Sirtoli, A.E., Silva, C.R., Botelho, M.F., 2013. Soil prediction using artificial neural networks and topographic attributes. Geoderma 195, 165-172.

Stevens, F., Bogaert, P., Van Oost, K., Doetterl, S., Van Wesemael, B., 2014. Regional-scale characterization of the geomorphic control of the spatial distribution of soil organic carbon in cropland. Eur J Soil Sci 65(4), 539-552.

Sun, W., Minasny, B., McBratney, A., 2012. Analysis and prediction of soil properties using local regression-kriging. Geoderma 171, 16-23.

Suuster, E., Ritz, C., Roostalu, H., Kolli, R., Astover, A., 2012. Modelling soil organic carbon concentration of mineral soils in arable land using legacy soil data. Eur J Soil Sci 63(3), 351-359.

Taghizadeh-Mehrjardi, R., Minasny, B., Sarmadian, F., Malone, B.P., 2014. Digital mapping of soil salinity in Ardakan region, central Iran. Geoderma 213, 15-28.

Tanikawa, T., Yamashita, N., Aizawa, S., Ohnuki, Y., Yoshinaga, S., Takahashi, M., 2013. Soil sulfur content and its spatial distribution in a small catchment covered by volcanic soil in the montane zone of central Japan. Geoderma 197, 1-8.

Taylor, J.A., Jacob, F., Galleguillos, M., Prevot, L., Guix, N., Lagacherie, P., 2013. The utility of remotely-sensed vegetative and terrain covariates at different spatial resolutions in modelling soil and watertable depth (for digital soil mapping). Geoderma 193, 83-93.

Vasques, G.M., Grunwald, S., Comerford, N.B., Sickman, J.O., 2010. Regional modelling of soil carbon at multiple depths within a subtropical watershed. Geoderma 156(3-4), 326-336.

Vohland, M., Besold, J., Hill, J., Frund, H.C., 2011. Comparing different multivariate calibration methods for the determination of soil organic carbon pools with visible to near infrared spectroscopy. Geoderma 166(1), 198-205.

Vohland, M., Ludwig, M., Thiele-Bruhn, S., Ludwig, B., 2014. Determination of soil properties with visible to near- and mid-infrared spectroscopy: Effects of spectral variable selection. Geoderma 223, 88-96.

Watt, M.S., Palmer, D.J., 2012. Use of regression kriging to develop a Carbon:Nitrogen ratio surface for New Zealand. Geoderma 183, 49-57.

Wetterlind, J., Piikki, K., Stenberg, B., Soderstrom, M., 2015. Exploring the predictability of soil texture and organic matter content with a commercial integrated soil profiling tool. Eur J Soil Sci 66(4), 631-638.

Wijewardane, N.K., Ge, Y.F., Morgan, C.L.S., 2016. Moisture insensitive prediction of soil properties from VNIR reflectance spectra based on external parameter orthogonalization. Geoderma 267, 92-101.

Wilford, J., de Caritat, P., Bui, E., 2015. Modelling the abundance of soil calcium carbonate across Australia using geochemical survey data and environmental predictors. Geoderma 259, 81-92.

Winowiecki, L., Vagen, T.G., Huising, J., 2016. Effects of land cover on ecosystem services in Tanzania: A spatial assessment of soil organic carbon. Geoderma 263, 274-283.
