# Supplementary material for: Generalizing soil properties in geographic space: Approaches used and ways forward
Source: PLoS One. 2018 Dec 21;13(12):e0208823. doi: 10.1371/journal.pone.0208823 (PMC6303050; doi:10.1371/journal.pone.0208823)
Supplement: S3 Table — Total number of studies for a. each type of approach (GSA: geostatistical approaches, PSA: predictive statistical approaches, HA: hybrid approaches); b. soil properties category and percentage of analyses used in each approach that obtained the highest spatialization performance value; c. study area extent category and percentage of analyses used in each approach that obtained the highest spatialization performance value; d. sample density category and percentage of analyses used in each approach that obtained the highest spatialization performance value. (DOCX) [file pone.0208823.s004.docx]

**S3 Table - The first group: comparative studies****.** Total number of studies for a. each type of approach (GSA: geostatistical approaches, PSA: predictive statistical approaches, HA: hybrid approaches); b. soil properties category and percentage of analyses used in each approach (geostatistical, predictive statistical, and hybrid) that obtained the highest spatialization performance value; c. study area extent category and percentage of analyses used in each approach (geostatistical, predictive statistical, and hybrid) that obtained the highest spatialization performance value; d. sample density category and percentage of analyses used in each approach (geostatistical, predictive statistical, and hybrid) that obtained the highest spatialization performance value.

| 1. **Approaches** | **GSA** | **PSA** | **HA** |
| --- | --- | --- | --- |
| **Number of analyses in total** | 9 | 38 | 31 |

| **b.** | **Numbers of analyses in total** | **Percentage of analyses** | | |
| --- | --- | --- | --- | --- |
|  |  | **GSA** | **PSA** | **HA** |
| **Grain size distr.** | 33 | 15% | 52% | 33% |
| **Org. carbon** | 22 | 14% | 50% | 36% |
| **Chem. prop.** | 13 | 0% | 62% | 38% |
| **Nitrogen** | 4 | 0% | 25% | 75% |
| **Exch. bases and ions** | 2 | 0% | 0% | 100% |
| **Other elements** | 2 | 50% | 0% | 50% |
| **Gen. descriptors** | 1 | 0% | 100% | 0% |
| **Potassium** | 1 | 0% | 0% | 100% |

| **c.** | **Numbers of analyses in total** | **Percentage of analyses** | | |
| --- | --- | --- | --- | --- |
|  |  | **GSA** | **PSA** | **HA** |
| **<1 km^2^** | 0 | 0% | 0% | 0% |
| **1.1-10 km2** | 4 | 25% | 25% | 50% |
| **11-100 km2** | 0 | 0% | 0% | 0% |
| **101-1000 km2** | 25 | 24% | 48% | 28% |
| **1001-10000 km2** | 10 | 10% | 0% | 90% |
| **>10000 km2** | 37 | 0% | 68% | 32% |

| **d.** | **Numbers of analyses in total** | **Percentage of analyses** | | |
| --- | --- | --- | --- | --- |
|  |  | **GSA** | **PSA** | **HA** |
| **<0.1/km2** | 45 | 2% | 56% | 42% |
| **0.11-1/km2** | 26 | 23% | 46% | 31% |
| **1.1-10/km2** | 1 | 0% | 0% | 100% |
| **10.1-100/km2** | 4 | 25% | 25% | 50% |
| **>100/km2** | 0 | 0% | 0% | 0% |
