# Supplementary material for: Generalizing soil properties in geographic space: Approaches used and ways forward
Source: PLoS One. 2018 Dec 21;13(12):e0208823. doi: 10.1371/journal.pone.0208823 (PMC6303050; doi:10.1371/journal.pone.0208823)
Supplement: S4 Table — Total number of studies for: a. each type of approach (GSA: geostatistical approaches, PSA: predictive statistical approaches, HA: hybrid approaches); b. each soil properties category and percentage of analyses used in each approach; c. each study area extent category and percentage of analyses used in each approach; d. each sample density category and percentage of analyses used in each approach; e. each altitudinal range category and percentage of analyses used in each approach; f. each year considered in the review and percentage of analyses used in each approach; g. continent and percentage of analyses used in each approach. (DOCX) [file pone.0208823.s005.docx]

**S4 Table - The second group of studies: on the use frequency of soil mapping approaches.** Total number of studies for: a. each type of approach (GSA: geostatistical approaches, PSA: predictive statistical approaches, HA: hybrid approaches); b. each soil properties category and percentage of analyses used in each approach; c. each study area extent category and percentage of analyses used in each approach; d. each sample density category and percentage of analyses used in each approach; e. each altitudinal range category and percentage of analyses used in each approach; f. each year considered in the review and percentage of analyses used in each approach; g. continent and percentage of analyses used in each approach.

| **a.** | GSA | PSA | HA |
| --- | --- | --- | --- |
| **Percentage of studies in total** | 9.8% | 78.3% | 12% |

|  | **Numbers of analyses in total** | **Percentage of analyses** | | |
| --- | --- | --- | --- | --- |
| **b.** |  | **GSA** | **PSA** | **HA** |
| **Grain size distr.** | 29 | 18.18% | 81.82% | 0.00% |
| **Org. carbon** | 52 | 11.54% | 80.77% | 7.69% |
| **Chem. prop.** | 22 | 13.64% | 77.27% | 9.09% |
| **Nitrogen** | 9 | 11.11% | 77.78% | 11.11% |
| **Exch. bases and ions** | 16 | 12.50% | 81.25% | 12.50% |
| **Other elements** | 11 | 27.27% | 63.64% | 0.00% |
| **Gen. descriptors** | 15 | 0.00% | 100.00% | 0.00% |
| **Inorg. carbon** | 6 | 13.64% | 77.27% | 9.09% |
| **Potassium** | 10 | 20.00% | 80.00% | 0.00% |

|  | **Numbers of analyses in total** | **Percentage of analyses** | | |
| --- | --- | --- | --- | --- |
| **c.** |  | **GSA** | **PSA** | **HA** |
| **<1 km^2^** | 12 | 16.67% | 75.00% | 16.67% |
| **1.1-10 km2** | 4 | 0.00% | 75.00% | 25.00% |
| **11-100 km2** | 10 | 0.00% | 70.00% | 30.00% |
| **101-1000 km2** | 17 | 5.88% | 82.35% | 11.76% |
| **1001-10000 km2** | 9 | 11.11% | 55.56% | 33.33% |
| **>10000 km2** | 24 | 12.50% | 87.50% | 0.00% |

|  | **Numbers of analyses in total** | **Percentage of analyses** | | |
| --- | --- | --- | --- | --- |
| **d.** |  | **GSA** | **PSA** | **HA** |
| **<0.1/km2** | 24 | 16.67% | 75.00% | 8.33% |
| **0.11-1/km2** | 9 | 5.26% | 78.95% | 15.79% |
| **1.1-10/km2** | 5 | 0.00% | 80.00% | 20.00% |
| **10.1-100/km2** | 6 | 0.00% | 66.67% | 33.33% |
| **>100/km2** | 11 | 18.18% | 72.73% | 9.09% |

|  | **Numbers of analyses in total** | **Percentage of analyses** | | |
| --- | --- | --- | --- | --- |
| **e.** |  | **GSA** | **PSA** | **HA** |
| **0 m** | 5 | 16.67% | 75.00% | 16.67% |
| **1-100 m** | 4 | 0.00% | 75.00% | 25.00% |
| **101-500m** | 10 | 0.00% | 70.00% | 30.00% |
| **501-1000m** | 5 | 5.88% | 82.35% | 11.76% |
| **1001-2000** | 3 | 11.11% | 55.56% | 33.33% |
| **>2000m** | 4 | 12.50% | 87.50% | 0.00% |

|  | **Numbers of analyses in total** | **Percentage of analyses** | | |
| --- | --- | --- | --- | --- |
| f. |  | **GSA** | **PSA** | **HA** |
| **2010** | 11 | 18.18% | 63.64% | 18.18% |
| **2011** | 5 | 0.00% | 100.00% | 0.00% |
| **2012** | 18 | 5.56% | 66.67% | 27.78% |
| **2013** | 12 | 16.67% | 83.33% | 0.00% |
| **2014** | 24 | 8.33% | 75.00% | 8.33% |
| **2015** | 11 | 9.09% | 81.82% | 9.09% |
| **2016** | 10 | 0% | 90% | 10% |

|  | **Numbers of analyses in total** | **Percentage of analyses** | | |
| --- | --- | --- | --- | --- |
| g. |  | **GSA** | **PSA** | **HA** |
| **Africa** | 3 | 0.00% | 100.00% | 0.00% |
| **Asia** | 16 | 12.50% | 81.25% | 6.25% |
| **Europe** | 41 | 7.32% | 80.49% | 12.20% |
| **Latin America** | 7 | 28.57% | 71.43% | 0.00% |
| **North America** | 14 | 14.29% | 71.43% | 14.29% |
| **Oceania** | 10 | 0.00% | 70.00% | 30.00% |
